# A mechanochemical approach to access the proline–proline diketopiperazine framework

**DOI:** 10.3762/bjoc.13.217

**Published:** 2017-10-19

**Authors:** Nicolas Pétry, Hafid Benakki, Eric Clot, Pascal Retailleau, Farhate Guenoun, Fatima Asserar, Chakib Sekkat, Thomas-Xavier Métro, Jean Martinez, Frédéric Lamaty

**Affiliations:** 1 Institut des Biomolécules Max Mousseron (IBMM), UMR 5247, CNRS, Université de Montpellier, ENSCM, Campus Triolet, Place Eugène Bataillon, 34095 Montpellier Cedex 5, France; 2 Laboratory of Chemistry Biology Applied to the Environment, Faculty of Sciences, Moulay Ismail University BP: 11201 Zitoune Meknès, Morocco; 3 Institut Charles Gerhardt, UMR 5253 CNRS-UM-ENSCM, Université de Montpellier, Place Eugène Bataillon, cc 1501, 34095 Montpellier Cedex 5, France; 4 Institut de Chimie des Substances Naturelles, CNRS UPR 2301, Université Paris-Saclay, 1 Avenue de la Terrasse, 91198 Gif-sur-Yvette, France

**Keywords:** ball mill, DFT calculations, diketopiperazine, mechanochemistry, pyrrolidine

## Abstract

Ball milling was exploited to prepare a substituted proline building block by mechanochemical nucleophilic substitution. Subsequently, the mechanocoupling of hindered proline amino acid derivatives was developed to provide proline–proline dipeptides under solvent-free conditions. A deprotection–cyclization sequence yielded the corresponding diketopiperazines that were obtained with a high stereoselectivity which could be explained by DFT calculations. Using this method, an enantiopure disubstituted Pro–Pro diketopiperazine was synthesized in 4 steps, making 5 new bonds using a ball mill.

## Introduction

2,5-Diketopiperazines (DKPs) are heterocyclic structures, usually derived from dipeptides, which find many applications in chemistry and biology, and have attracted attention in the last years [1–2]. The diketopiperazine backbone can be found in many natural products exhibiting various biological activities [3]. Consequently, medicinal chemists have used DKPs extensively as a synthetic platform, easily synthesized and stereochemically controlled, for the preparation of small bioactive molecules [4–5]. DKPs have also been considered as chiral auxiliaries in asymmetric synthesis [6]. Furthermore, the rigidity of the DKPs is a unique feature, used for the preparation of biologically active peptides and peptidomimetics [7], for applications in organocatalysis [8–10], and for the preparation of novel materials [11–12].

An interesting sub-family of these compounds are DKPs derived from the amino acid proline and its analogues, which provide a useful rigid structure. During the course of our project on the exploitation of dimethyl dibromoadipate as a synthon to access original molecules [13–14], we thought that it could provide an original access to the DKP Pro–Pro framework. More specifically, this type of framework has been used as a scaffold for the preparation of small compound libraries [15].

**Scheme 1:**
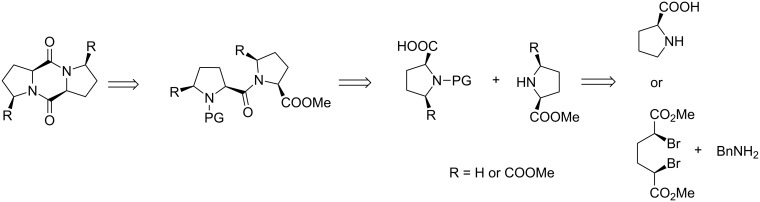
Retrosynthesis of the Pro–Pro DKP framework.

The Pro–Pro diketopiperazine can be prepared directly by dimerization of unprotected proline in a one-pot transformation, generally under harsh conditions [16]. Good results were indeed reported, although this procedure gives access only to symmetrical products and can be detrimental for more fragile molecules such as substituted enantiomerically pure compounds. As shown by a retrosynthetic analysis (Scheme 1), a classical milder approach would consist in preparing first the dipeptide, followed by an intramolecular ester aminolysis. This strategy has been extensively used [1], involving milder conditions and provides access to unsymmetrical dipeptides and DKPs. Furthermore, substituted prolines could be obtained by nucleophilic substitution of benzylamine from dimethyl dibromoadipate, allowing the addition of functional groups on the Pro–Pro-based framework [17]. Recently, mechanochemistry has become a powerful synthetic technique for making new organic molecules [18–19]. In the course of this project, we applied mechanochemistry to a nucleophilic substitution and the efficient coupling of two proline residues.

## Results and Discussion

**Scheme 2:**
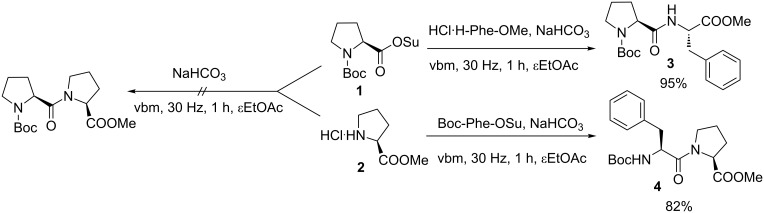
Coupling with *N*-hydroxysuccinimide-activated amino acids.

First we studied the preparation of simple Pro–Pro DKP as a model compound. The use of ball milling in peptide synthesis has drawn some attention in the recent years [20–28]. We took advantage of our extensive experience in peptide mechanosynthesis [20,23–25,27] to prepare the Pro–Pro dipeptide from the corresponding amino acid derivatives. We investigated the coupling of proline *N*-hydroxysuccinimide ester with proline methyl ester in a vibrating ball mill (vbm, Scheme 2) [23].

Surprisingly, while the coupling of various other amino acids previously used yielded the corresponding dipeptides [23], no reaction occurred in the case of the two prolines **1** and **2**, even by varying the reaction conditions. To verify the reactivity of either Boc–Pro–OSu (**1**) or H–Pro–OMe (**2**) in the mechanocoupling, we reacted HCl·H–Phe–OMe or Boc–Phe–OSu with respectively Boc–Pro–OSu and HCl·H–Pro–OMe. In both cases, the reaction proceeded smoothly to give good yields of dipeptides **3** and **4** (95% of Boc–Pro–Phe–OMe and 82% of Boc–Phe–Pro–OMe, respectively). Most probably, this method was less adapted to hindered amino acid derivatives such as proline.

As an alternative approach, we tested the optimal conditions developed previously for peptide mechanosynthesis [25], starting with unactivated amino acids together with a coupling agent. We had indeed reported two successful examples of couplings involving proline amino esters. The initial conditions, using 1-ethyl-3-(3-dimethylaminopropyl)carbodiimide (EDC), ethyl cyano(hydroxyimino)acetate (oxyma) in the presence of a base and a liquid additive, were adapted to the preparation of Z–Pro–Pro–OMe (**7**) and Boc–Pro–Pro–OMe (**8**, Table 1). It consisted in ball milling the two amino acid derivatives **5** or **6** with **2** in the presence of EDC (coupling agent), a base and a small amount [29] of EtOAc as liquid grinding assistant. The role of oxyma was mainly to suppress amino acid epimerization during the coupling, a limited problem in the case of proline. Consequently, our first experiments did not involve this reagent (Table 1, entries 1–3). Gratifyingly, the initial results showed that this method was adequate to prepare the Pro–Pro dipeptide **7** albeit in fair yield (Table 1, entry 1). Adding more starting material **6** (Table 1, entry 2) and changing the base (Table 1, entry 3) did not provide much improvement. Finally supplementing the reaction mixture with oxyma (Table 1, entries 4–7) increased the yield up to 85–90% depending on the protection on the proline nitrogen (Boc or Z). Both of the bases gave similar yields (Table 1, entry 5 vs 7 and entry 4 vs 6). Eventually, as proposed before [25], NaH_2_PO_4_ was preferred since it would avoid a potential pressure build-up (release of CO_2_) which could occur with NaHCO_3_. Noteworthy, no epimerization could be detected by NMR or HPLC analyses.

**Table 1:** Optimization of the Pro-Pro coupling^a^.

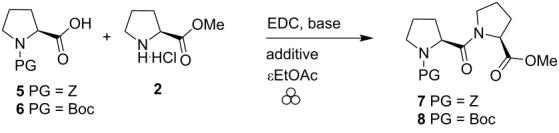						

Entry	PG	equiv of **5** or **6**	Base (equiv)	Activating agent (equiv)	Reaction time	Yield (%)

1	Boc	1.2	NaHCO_3_ (3)	EDC (1.2)	1 h	65
2	Boc	1.2 + 0.5	NaHCO_3_ (3)	EDC (1.5)	2 × 45 min	68
3	Boc	1.2 + 0.5	NaH_2_PO_4_ (3)	EDC (1.5)	2 × 45 min	66
4	Boc	1.2	NaHCO_3_ (4)	EDC/oxyma (1.2)	1 h	78
5	Z	1.2	NaHCO_3_ (4)	EDC/oxyma (1.2)	1 h	90
6	Boc	1.2	NaH_2_PO_4_ (4)	EDC/oxyma (1.2)	1 h	85
7	Z	1.2	NaH_2_PO_4_ (4)	EDC/oxyma (1.2)	1 h	88

^a^Reactions performed under air, in a vibrating ball mill (vbm) at 30 Hz with EtOAc (as a liquid grinding assistant).

**Scheme 3:**
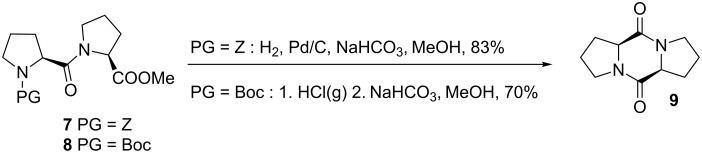
Synthesis of Pro–Pro DKP.

Both peptides **7** and **8** were then deprotected and cyclized into the corresponding diketopiperazine **9**. Palladium-catalyzed hydrogenolysis of the Z group of **7**, in the presence of NaHCO_3_, in MeOH, provided the DKP **9** in 83% yield. Compound **8** was deprotected with gaseous HCl, and the resulting dipeptide was cyclized in the presence of NaHCO_3_, in MeOH, yielding 70% of **9** (Scheme 3).

Then, as proposed above, we expanded this method to the preparation of substituted Pro–Pro DKPs. For this purpose, we considered using dimethyl (2*R*,5*S*)-pyrrolidine-2,5-dicarboxylate (*cis*-**11**) as a building block in the synthesis of dipeptides and diketopiperazines. This building block was used in a very limited number of cases for the formation of DKP in combination with an amino acid derivative [30–31]. Original preparative conditions of the protected compound **11** consisted in performing a nucleophilic substitution of benzylamine with meso dimethyl-2,5-dibromohexanedioate (**10**) in benzene or toluene as solvent, yielding two diastereomers *cis*-**11** (meso) and *trans-***11** (racemic), which could be separated by crystallization or column chromatography [17,32–33]. Trying to avoid as much as possible the use of (toxic) solvents, we considered extending the known nucleophilic substitution in a ball mill [34–41] to this reaction system (Table 2).

**Table 2:** Optimization of the substitution reaction.

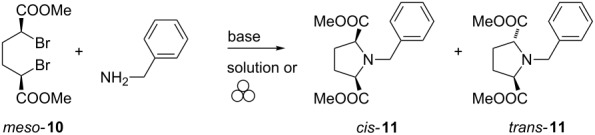					

Entry	equiv BnNH_2_	Base (equiv)	Conditions	Conversion*^a^*	*cis*/*trans-***11** ratio

1	3	–	toluene, 16 h, reflux	100	78:22
2	1	K_2_CO_3_ (1.2)	vbm, 1 h, 25 Hz	40	96:04
3	1	K_2_CO_3_ (3)	vbm, 1 h, 25 Hz	62	98:02
4	1.1	Cs_2_CO_3_ (3)	vbm, 1 h, 25 Hz	74	91:09
5	1.1	Cs_2_CO_3_ (3)	vbm, 1 h, 30 Hz	82	94:06
6	1.1	K_2_CO_3_ (3)	vbm, 1 h, 30 Hz^b^	49	98:02
7	1.1	Cs_2_CO_3_ (3)	vbm, 1 h, 30 Hz^b^	59	87:13
8	1.3	K_2_CO_3_ (2.2)	pbm, 2 h, 500 rpm^b^	97	97:03

^a^Measured by ^1^H NMR ^b^EtOAc was used as liquid grinding assistant.

For sake of comparison, we first performed the reaction between *meso*-**10** and benzylamine in toluene (Table 2, entry 1) providing a full conversion into the expected product **11** with a 78:22 *cis*/*trans* ratio. Then we studied the mechanosynthesis of these compounds (Table 2, entries 2–8), starting by mixing an equimolar amount of the starting materials together with a base (K_2_CO_3_) in a vibratory ball mill at 25 Hz (Table 2, entry 2). This resulted in a lower conversion compared to that obtained in solution. Using an excess of base increased the conversion to 62% (Table 2, entry 3). Switching to Cs_2_CO_3_ resulted in an increased conversion of 74% (Table 2, entry 4), further improved to 82% when the milling frequency was adjusted to 30 Hz (Table 2, entry 5). Adding EtOAc as liquid grinding assistant did not improve the conversion, with either K_2_CO_3_ or Cs_2_CO_3_ (Table 2, entries 6 and 7). Finally, we tested the planetary ball mill (pbm) with the advantage of its capacity to produce more material. In this case (Table 2, entry 8), using cheaper K_2_CO_3,_ full conversion was obtained and *cis*-**11** was isolated in 75% yield and a larger amount of *cis*-**11** could be prepared. Interestingly the *cis*/*trans* ratio (*cis*-**11**/*trans*-**11**) was different when the reaction was performed in solution (Table 2, entry 1) or in the ball mill (Table 2, entries 2–9) with a higher selectivity in the latter case [42].

**Scheme 4:**
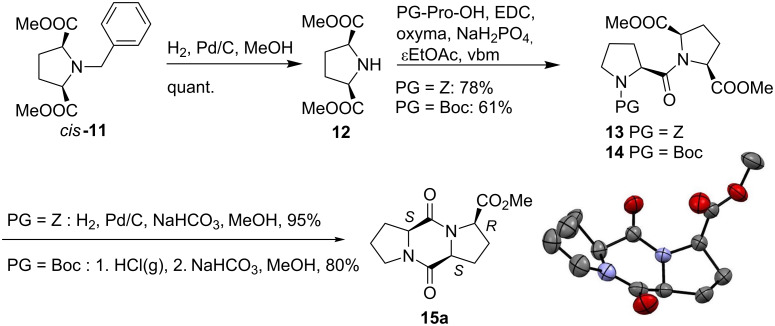
Synthesis of substituted Pro–Pro DKP **15a**.

With this building block in hands, the preparation of a variety of DKPs could be envisaged (Scheme 4).

**Scheme 5:**
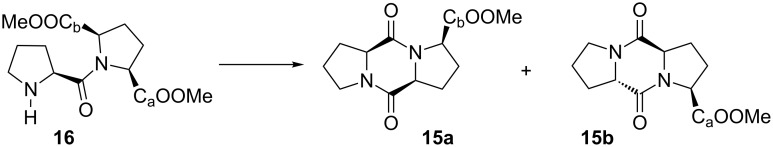
Potential isomers yielded by cyclization of **16**.

Pyrrolidine *cis*-**11** is an *N-*protected amino ester, which can be used in the synthesis of diketopiperazines by deprotecting either the amino group or the ester function. Hydrogenolysis of the benzyl group of *cis*-**11** provided the nitrogen-free pyrrolidine derivative **12** in excellent yield and purity after filtration of the catalytic system. **12** was engaged without further purification in a coupling reaction with Z-proline (**5**) and Boc-proline (**6**), in the solvent-free conditions described above. In both cases, the dipeptides **13** and **14** were obtained in good yields (78 and 61%, respectively). Deprotection followed by cyclization provided the corresponding diketopiperazine **15a** in 95% yield (from **13**) or 80% yield (from **14**). In this case, two carboxymethyl groups could participate in the cyclization providing two possible diastereomers **15a** and **15b** (Scheme 5).

To our delight, this stereodivergent cyclization was selective and only one diastereomer was obtained, as supported by analytical data. X-ray analysis of the product confirmed the stereochemistry of the three chiral centres and the structure of **15a**.

To shed more light on the origin of the selectivity observed in the deprotection–cyclization transformation, DFT calculations of the reaction mechanism have been carried out. DFT calculations were applied to the various pathways starting from the deprotected amine **16** and reaction pathways leading to either product **15a**, resulting from nucleophilic attack of the amine on C_a_, or to product **15b** resulting from attack on C_b_, were considered (Scheme 5).

**Figure 1:**
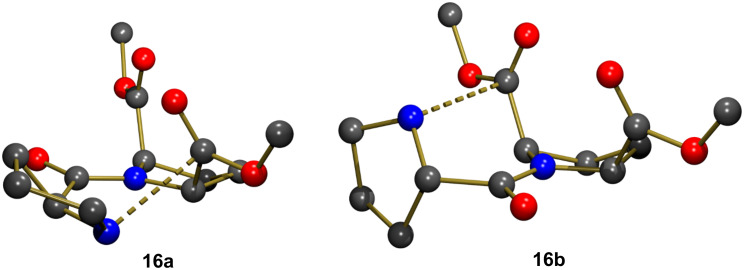
Optimized geometries for the two conformers presenting interactions with either C_a_ (**16a**) or C_b_ (**16b**). H atoms were omitted for clarity.

The first step was to study if there was any preferential interaction between the free nitrogen atom and either C_a_ or C_b_ before the C–N bond formation. Both optimized structures are shown in Figure 1, and compound **16a** is computed to be less stable than **16b** by Δ*G* = 2.7 kcal mol^−1^. The C···N bond distance is slightly shorter in **16b** (2.673 Å) than in **16a** (2.682 Å). Many attempts to locate a transition state structure for the C–N bond formation starting from either **16a** or **16b** failed. Even though the geometry optimizations were performed with implicit inclusion of the solvent influence (SMD model with methanol), the zwitterionic character developing in the C–N bond formation could not be stabilized. However, the protic methanol solvent could act both as a base to abstract the proton from the nitrogen atom, and as an acid to facilitate the C–OMe bond cleavage. Transition state structures with combined implicit (SMD model) and explicit inclusion of the solvent were thus searched for.

**Figure 2:**
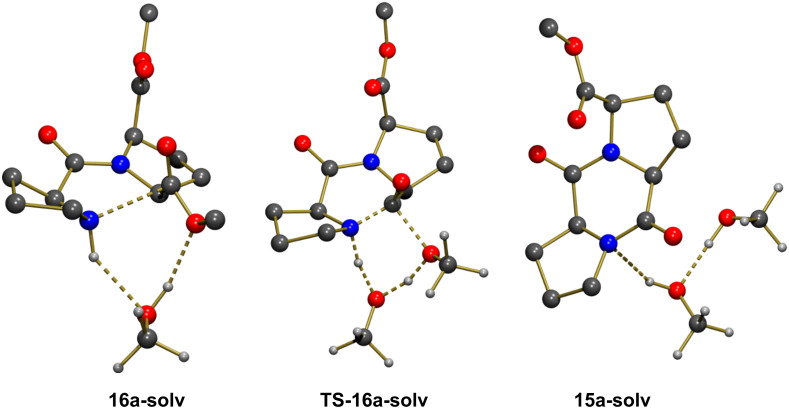
Optimized geometries of the extrema located along the pathway for formation of **15a** with explicit participation of one solvent molecule. Most H atoms were omitted for clarity.

The geometry of **16a** allowed the creation of a network of stabilizing interactions between an explicit methanol solvent molecule and both the N–H proton and the OMe group (N–H···O = 2.153 Å, H···OMe = 1.925 Å; see **16a-solv** in Figure 2). **16a-solv** was computed to be less stable than **16a** by Δ*G* = 9.3 kcal mol^−1^. This higher Gibbs free energy was due only to entropic factors as **16a-solv** was computed to be more stable than **16a** by Δ*E* = −4.0 kcal mol^−1^. Interestingly, upon interaction with an explicit methanol molecule the C···N distance in **16a-solv** had been reduced to 2.464 Å compared to a value of 2.682 Å in **16a**. A transition state structure, **TS-16a-solv**, corresponding to a concerted C–N bond formation and a C–OMe bond cleavage could be located (Figure 2). Table 3 collects selected bond distances associated to the transformation. In the transition state, the C–OMe bond cleavage was well advanced and the C–N bond formation was also almost complete. This indicated that the transformation was concerted and that the explicit methanol molecule only acted as a relay to accept the proton from the amine and to facilitate the departing of the methoxy group by transferring a proton. The activation energy from **16a-solv** was computed to be Δ*G*^#^ = 22.8 kcal mol^−1^, in good agreement with an easy reaction at room temperature. The reaction was strongly exoergic with ΔG = −17.3 kcal mol^−1^ and the geometry of **15a-solv** (Figure 2) had the stereochemistry expected for **15a** (Scheme 5).

**Table 3:** Selected bond distances (Å) for the structures optimized along the transformation **16a-solv**→**15a-solv**.

Bond	**16a-solv**	**TS-16a-solv**	**15a-solv**

N–H	1.077	1.167	2.310
NH···O	2.153	1.341	0.965
MeO–H	0.970	1.226	1.761
H···OMe	1.925	1.117	0.979
C–OMe	1.340	1.935	3.784
N–C	2.464	1.500	1.343

**Figure 3:**
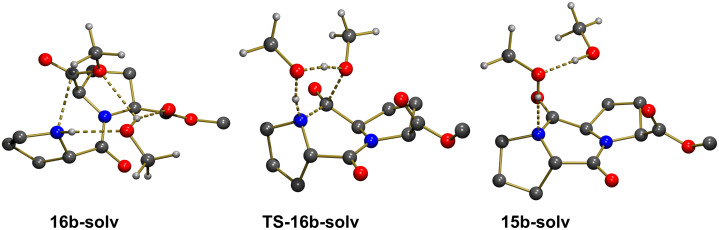
Optimized geometries of the extrema located along the pathway for formation of **15b** with explicit participation of one solvent molecule. Most H atoms were omitted for clarity.

The geometry of **16b** did not allow creating a similar network of H-bonding interactions when one explicit molecule of methanol was considered. The N–H bond is pointed in a direction of space remote from the methoxy group of the ester functionality. Rotation by 180° around the C–C bond of the ester led to a geometry in which a methanol molecule could interact with both groups as illustrated in **16b-solv** (Figure 3). This structure was computed to be more stable than **16a-solv** by Δ*G* = −3.8 kcal mol^−1^, probably because in addition to the expected H-bonds between N–H and O (N–H···O = 2.123 Å), and between O–H and OMe (H···OMe = 2.488 Å), there existed an additional H-bond with the other ester functionality (H···OC = 1.873 Å). However, despite the greater stability of **16b-solv**, the concerted formation of C–N and cleavage of the C–OMe bond through **TS-16b-solv** was associated to a higher activation barrier with Δ*G*^#^ = 30.0 kcal mol^−1^ and a less exoergic reaction (Δ*G* = −2.6 kcal mol^−1^). Selected bond distances in Table 4 clearly show that the formation of C–N and cleavage of C–O are both well advanced in **TS-16b-solv**, similarly to the situation observed in **TS-16a-solv**. The essential difference was the significant longer C···N distance in **16b-solv** (2.625 Å vs 2.464 Å in **16a-solv**), and the longer H-bond between the methanol molecule and the methoxy group in **16b-solv** (2.488 Å) compared to that observed in **16a-solv** (1.925 Å). The origin of these differences lied in the presence of an H-bond between the methanol molecule and the carbonyl group of the other ester functionality. This interaction stabilized a geometry with a longer C···N distance, and destabilized the transition state structure as it needed to be lost in **TS-16b-solv** (H···OC = 3.326 Å vs 1.873 Å in **16b-solv**).

**Table 4:** Selected bond distances (Å) for the structures optimized along the transformation **16b-solv**→**15b-solv**.

Bond	**16b-solv**	**TS-16b-solv**	**15b-solv**

N–H	1.018	1.165	2.014
NH···O	2.123	1.342	0.971
MeO–H	0.973	1.212	1.751
H···OMe	2.488	1.182	0.979
C–OMe	1.326	1.940	3.333
N–C	2.625	1.521	1.365

**Figure 4:**
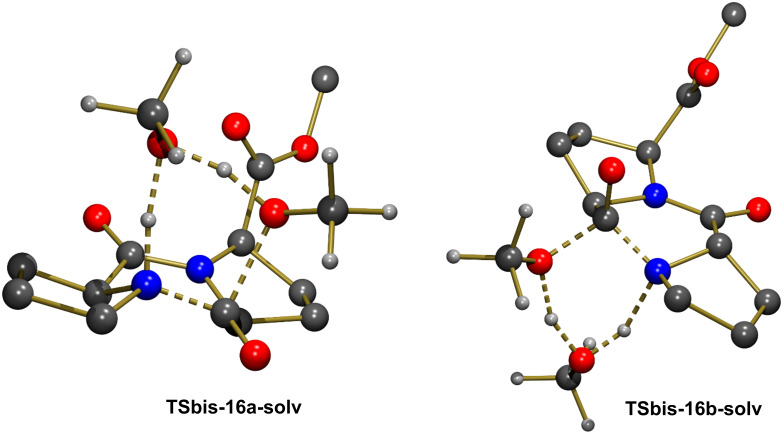
Optimized geometries for the transition states associated to alternate position of the methanol molecule. Most H atoms were omitted for clarity.

There was thus a significant energetic preference for the formation of **15a** with respect to **15b** with a ΔΔ*G*^#^ = 7.3 kcal mol^−1^. However, the positions of the methanol molecule in **TS-16a-solv** and **TS-16b-solv** were significantly different, and this could be the origin of the stability of the former. Therefore a transition state structure leading to **15b** with the methanol molecule in an “exo” position was optimized (**TSbis-16b-solv**, Figure 4). This transition state was less stable than **TS-16b-solv** by 2.9 kcal mol^−1^. Alternatively, a transition state structure leading to **15a** with a methanol molecule in an “endo” position was located (**TSbis-16a-solv**, Figure 4). This structure was computed to be less stable than **TS-16a-solv** by 3.9 kcal mol^−1^. The calculations thus clearly indicated that there was a low lying pathway for the formation of **15a** consisting in a concerted C–N bond formation and C–OMe bond cleavage mediated by a solvent methanol molecule acting as both a proton acceptor from N–H and a proton donor to OMe. All the alternative pathways were associated to transition states lying at significantly higher energy not to be observed experimentally. This was in agreement with the experimental formation of only **15a**.

**Scheme 6:**
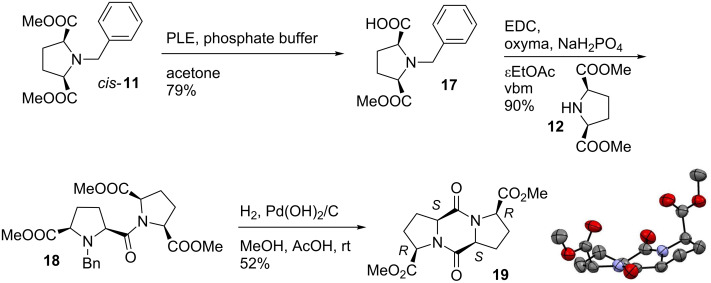
Synthesis of diketopiperazine **19**.

As mentioned above, another possibility to exploit *meso* pyrrolidine *cis*-**11** would be to desymmetrize [43] the ester functions by selective hydrolysis. The corresponding carboxylic acid could then be engaged in a peptide coupling. Pig liver esterase (PLE)-catalyzed enzymatic hydrolysis of meso *cis*-**11** provided selectively the *N*-protected amino acid **17** as one enantiomer [33,44–45]. Mechanocoupling of **17** with pyrrolidine **12** provided the dipeptide **18** in excellent yield. Removal of the benzyl group by hydrogenation in the presence of Pd(OH)_2_/C followed by cyclization provided unprecedented DKP **19** in 52% yield. In this case again, spectral data and X-ray analysis showed the selective formation of diketopiperazine **19** as only one isomer (Scheme 6).

## Conclusion

In summary, we have developed an efficient synthesis of two enantiopure substituted diketopiperazines based on the proline–proline framework. The synthetic schemes included two key reactions, which were performed under mechanochemical conditions, including a peptide coupling leading to the formation of Pro–Pro dipeptides, and a nucleophilic substitution furnishing substituted proline derivatives. The diastereoselective cyclization, which was clearly supported by DFT calculations is noteworthy. Further developments and applications of these scaffolds are currently underway.

## Supporting Information

Experimental procedures and characterization of new compounds, X-ray data including CCDC numbers and CIF files.

File 1 Experimental part.

File 2 Crystallographic data.

File 3 X-ray of meso-**10**.

File 4 X-ray of **15a**.

File 5 X-ray of **19**.
