# Supplementary material for: A mechanochemical approach to access the proline–proline diketopiperazine framework
Source: Beilstein J Org Chem. 2017 Oct 19;13:2169–78. doi: 10.3762/bjoc.13.217 (PMC5669228; doi:10.3762/bjoc.13.217)
Supplement: File 1 — Experimental part. [file Beilstein_J_Org_Chem-13-2169-s001.pdf]

Supporting Information

for

A mechanochemical approach to access the proline–proline diketopiperazine framework

Nicolas Pétry[1], Hafid Benakki[1,2], Eric Clot[3], Pascal Retailleau[4], Farhate Guenoun[2], Fatima Asserar[2], Chakib Sekkat[2], Thomas-Xavier Métro*[1], Jean Martinez[1] and Frédéric Lamaty*[1]

Address: [1]Institut des Biomolécules Max Mousseron (IBMM), UMR 5247, CNRS, Université de Montpellier, ENSCM, Campus Triolet, Place Eugène Bataillon, 34095 Montpellier Cedex 5, France, [2]Laboratory of Chemistry Biology Applied to the Environment, Faculty of Sciences, Moulay Ismail University BP: 11201 Zitoune Meknès, Morocco, [3]Institut Charles Gerhardt, UMR 5253 CNRS-UM-ENSCM, Université de Montpellier, Place Eugène Bataillon, cc 1501, 34095 Montpellier Cedex 5, France and [4]Institut de Chimie des Substances Naturelles, CNRS UPR 2301, Université Paris-Saclay, 1 Avenue de la Terrasse, 91198 Gif-sur-Yvette, France

Email: Thomas-Xavier Métro - thomas-xavier.metro@umontpellier.fr; Frédéric Lamaty - frederic.lamaty@umontpellier.fr

*Corresponding author

Experimental part

General. All reagents were purchased from Sigma-Aldrich, Fluka, Novabiochem or Senn Chemicals and were used without further purification. The milling treatments were carried out either in a vibrating Retsch Mixer Mill 200 or 400 (vbm) or in a Fritsch Pulverisette 7 Planetary Mill (pbm). The milling load (ML) is defined as the sum of the mass of reagents per free volume in the jar. The free volume is the volume of the jar minus the volume of the ball. ML is expressed in mg/mL. η is the ratio of added liquid volume to the mass of reagents. It is expressed in µL/mg. NMR spectra were recorded on a Bruker AVANCE DPX 300 MHz, a Bruker AVANCE III 400 MHz, a Bruker AVANCE III 500 MHz or a Bruker AVANCE III 600 MHz spectrometer and are reported in ppm using solvent as an internal standard ($CDCl_3$ at 7.26 ppm). Data are reported as s = singlet, d = doublet, t = triplet, m = multiplet or overlap of non-equivalent resonances; integration; coupling constant in Hz. ^{13}C NMR spectra are reported in ppm using the solvent as an internal standard ($CDCl_3$ at 77.16 ppm).

Analytical high performance liquid chromatography was performed on a Waters Millenium 717 that was equipped with an autosampler and had a variable wavelength diode detector. A Chromolith RP18 col- umn (50 × 4.6 mm) was employed, and a flow rate of 5 mL min^{-1} was used with a linear gradient of CH_3CN in water of 0–100% (0.1% TFA) over 4.5 min. Flash chromatography was performed by using prepacked silica columns on a Biotage® IsoleraTM Four system.
High resolution LC–UV–MS analyses were performed on UPLC Acquity H-Class from Waters with Kinetex C18 100 Å 2.1 × 2.6 mm column from Phenomenex hyphenated to a Synapt G2-S mass spectrometer with a dual ESI source from Waters. UV chromatograms were recorded with a PDA detector from 200 to 400 nm. Analyses were carried out with the column oven at 25 °C, with a flow rate of 500 µl min^{-1} and an injection volume of 1 µL. Elution solvents used were water and acetonitrile each supplemented with 0.1% formic acid. Gradient elution was performed from 0% to 100% of acetonitrile 0.1% formic acid in 12 min. Mass spectra were recorded in positive mode from 100 to 1500 Da with a capillary voltage of 3000 V and a cone voltage of 30 V. Source and desolvation temperatures were respectively 140 °C and 450 °C.

Computational details. Geometry optimizations have been performed with the Gaussian 09 package[1] at the wB97XD level of hybrid density functional theory,[2] with inclusion of both implicit solvent influence using the SMD model for methanol,[3] and the D3(bj) corrections in the optimization process.[4,5] The atoms were represented by a def2-svp basis set.[6] To gain a better estimate of the energies, single point calculations on the solvent-optimized geometries were carried out using a def2-qvvpp basis set for all the atoms.[7] All energies reported in the present work are Gibbs free energies obtained by summing the energy (including D3 corrections) computed with the def2-qzvpp basis sets and the Gibbs contribution as deduced on the solvent-optimized geometries with the def2-svp basis sets at 298 K and 1 atm.

Boc-Pro-Phe-OMe **(3)**

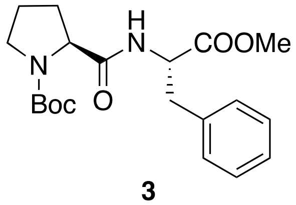

3

Procedure:[8] Boc-L-proline *N*-hydroxysuccinimide ester (**1**, 130 mg, 0.42 mmol), L-phenylalanine methyl ester hydrochloride (90 mg, 0.42 mmol, 1.0 equiv), $NaHCO_3$ (84 mg, 0.62 mmol, 1.5 equiv) and EtOAc (330 μL, η = 1.20 μL.mg^{-1}) as liquid assistant were introduced in a 10 mL stainless steel grinding jar with one stainless steel ball (10 mm diameter). The jar was closed and placed within the vibratory ball-mill for 1 h at 30 Hz. The residue was then dissolved in EtOAc and water, the layers were separated and the aqueous one was extracted once with EtOAc. The combined organic phases were washed twice with 1 N aqueous NaOH solution, twice with 1 N aqueous HCl solution and once with brine. After drying over $MgSO_4$, filtration and evaporation under vacuum, the dipeptide Boc-Pro-Phe-OMe **3** (150 mg, 0.40 mmol, 95%) was obtained as a white solid. Analytical data were in agreement with those reported in the literature. [9]

TLC: Rf = 0.56 (cyclohexane/EtOAc 50/50) (ninhydrin)
^{1}H NMR (400 MHz, $CDCl_3$) δ (ppm): (minor rotamer in parentheses) 1.42 (s, 9H), 1.77 (br s, 2H), 1.99 (m, 1H), 2.26 (br s, 1H), 3.01 (dd, 1H, *J* = 13.9 Hz, 7.0 Hz), 3.19 (dd, 1H, *J* = 13.9 Hz, 5.6 Hz), 3.29 (3.40) (m, 2H), 3.72 (s, 3H), 4.19 (4.28) (br s, 1H), 4.86 (br s, 1H), 6.46 (br s, 1H), 7.09 (d, 2H, *J* = 7.1 Hz), 7.20-7.30 (m, 3H).
^{13}C NMR (100 MHz, CDCl3) δ (ppm): (minor rotamer in parentheses) 23.5 (24.6), 28.4 (30.8), 38.2, 47.0, 52.4, 52.7 (53.4), 60.0 (61.1), 80.5 (80.9), 127.1 (127.3), 128.5 (128.7), 129.3, 135.9 (136.2), 154.7 (155.8), 171.8, 172.3.

Boc-Phe-Pro-OMe **(4)**

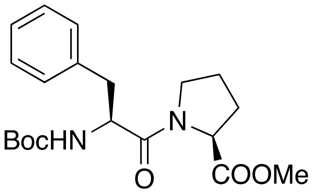

4

Procedure:[8] Boc-L-phenylalanine *N*-hydroxysuccinimide ester (140 mg, 0.38 mmol), L-proline methyl ester hydrochloride **2** (63 mg, 0.38 mmol, 1.0 equiv), $NaHCO_3$ (48 mg, 0.57 mmol, 1.5 eq) and EtOAc (270 μL, η = 1.07 μL.mg^{-1}) as liquid assistant were introduced in a 10 mL stainless steel grinding jar with one stainless steel ball (10 mm diameter). The jar was closed and submitted to vibration milling for 1 h at 30 Hz. The residue was then dissolved in EtOAc and water, the layers were separated and the aqueous phase was extracted once with EtOAc. The combined organic phases were washed twice with 1 N aqueous NaOH solution, twice

with 1 N aqueous HCl solution and once with brine. After drying over $MgSO_4$, filtration and evaporation under vacuum, the dipeptide Boc-Phe-Pro-OMe **4** (118 mg, 0.31 mmol, 82%) was obtained as a colorless wax. Analytical data were in agreement with those reported in the literature.[10]

TLC: Rf = 0.55 (cyclohexane/EtOAc 50/50) (ninhydrin)
^{1}H NMR (300 MHz, $CDCl_3$) δ (ppm): (minor rotamer in parentheses) 1.38 (1.42) (s, 9H), 1.84-2.00 (1.60-1.76) (m, 3H), 2.16 (m, 1H), 2.91 (m, 1H), 3.06 (m, 1H), 3.19 (3.31) (m, 1H), 3.59 (m, 1H), 3.74 (3.69) (s, 3H), 4.50 (4.42) (dd, 1H, *J* = 4.2 Hz, 8.1 Hz), 4.65 (m, 1H), 5.34 (5.45) (d, 1H, *J* = 8.5 Hz), 7.19-7.34 (m, 5H).
^{13}C NMR (75 MHz, CDCl3) δ (ppm): (minor rotamer in parentheses) 24.9 (22.3), 28.4 (29.1), 39.2 (41.2), 46.9 (46.1), 52.3 (52.8), 53.3 (53.9), 59.0, 79.7 (79.6), 126.8 (127.1), 128.4 (128.6), 129.8 (129.4), 136.4 (136.6), 155.3 (154.8), 170.9, 172.4 (171.9).

Z-Pro-Pro-OMe (7)

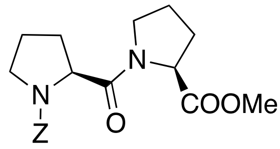

7

Procedure:[11] *(S)*-proline methyl ester hydrochloride (**2**, 73 mg, 0.44 mmol), (*S*)-*N*-benzyloxy-carbonylproline **5** (132 mg, 0.53 mmol, 1.2 equiv), $NaHCO_3$ (150 mg, 1.76 mmol, 4 equiv), oxyma (75 mg, 0.53 mmol, 1.2 equiv), EDCI (95 μL, 0.53 mmol, 1.2 equiv) and EtOAc (260 μL, η = 0.50 μL mg^{-1}) as liquid assistant were introduced in a 10 mL stainless steel grinding jar with one stainless steel ball (10 mm diameter). The jar was closed and submitted to vibration milling for 1 h at 30 Hz. The residue was then dissolved in EtOAc and water, the layers were separated and the organic one was washed with 1 N aqueous HCl solution, saturated aqueous $NaHCO_3$ solution and brine. After drying over $MgSO_4$, filtration and evaporation under vacuum, the dipeptide **7** (154 mg, 0.41 mmol, 90%) was obtained as a colorless wax and engaged in the next step without further purification. Analytical data were in agreement with those reported in the literature.[12]

TLC: Rf = 0.50 (EtOAc) (UV, ninhydrine)
^{1}H NMR (400 MHz, $CDCl_3$) δ (ppm): (2 rotamers) 1.62-2.25 (m, 8H), 3.33-3.84 (m, 4H), 3.70, 3.71 (s, 3H), 4.35-4.44 (m, 1H), 4.54-4.61 (m, 1H), 4.96-5.19 (m, 2H), 7.26-7.36 (m, 5H).
^{13}C NMR (100 MHz, CDCl3) δ (ppm): (2 rotamers) 23.7, 24.4, 24.9, 25.1, 28.7, 28.9, 29.2, 30.3, 46.6, 46.7, 46.8, 47.4, 52.3, 52.3, 57.6, 58.3, 58.7, 58.9, 67.0, 67.3, 127.9, 128.0, 128.1, 128.2, 128.5, 128.6, 136.8, 136.9, 154.2, 155.1, 171.1, 171.4, 172.7, 173.0.
HRMS ESI-(+) calcd. for $C_{19}H_{25}N_2O_5$ $[M+H]^+$ 361.1763, found 361.1765.

Boc-Pro-Pro-OMe (8)

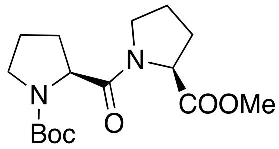

8

Procedure:[11] *(S)*-proline methyl ester hydrochloride **2** (110 mg, 0.66 mmol), (*S*)-*N*-*tert*-butyloxycarbonylproline **6** (172 mg, 0.80 mmol, 1.2 equiv), NaH_2PO_4 (320 mg, 2.66 mmol, 4 equiv), oxyma (114 mg, 0.80 mmol, 1.2 equiv), EDCI (142 μL, 0.80 mmol, 1.2 equiv) and EtOAc (450 μL, η = 0.53 $\mu L.mg^{-1}$) as liquid assistant were introduced in a 15 mL PTFE grinding jar with one stainless steel ball (10 mm diameter). The jar was closed and submitted to vibration milling for 1 h at 30 Hz. The residue was then dissolved in EtOAc and water, the layers were separated and the aqueous one was extracted once with EtOAc. The combined organic phases were washed with 1 N aqueous HCl solution, saturated aqueous $NaHCO_3$ solution and brine. After drying over $MgSO_4$, filtration and evaporation under vacuum, the dipeptide **8** (183 mg, 0.66 mmol, 85%) was obtained as a colorless wax and engaged in the next step without further purification. Analytical data were in agreement with those reported in the literature.[13]

TLC: Rf = 0.47 (EtOAc) (ninhydrin)
^{1}H NMR (400 MHz, $CDCl_3$) δ (ppm): (2 rotamers) 1.37, 1.43 (s, 9H), 1.77-2.22 (m, 8H), 3.34-3.48 (m, 1H), 3.50-3.65 (m, 2H), 3.69, 3.70 (s, 3H), 3.70-3.79 (m, 1H), 4.36-4.58 (m, 2H).
^{13}C NMR (100 MHz, CDCl3) δ (ppm): (2 rotamers) 23.7, 24.2, 25.1, 25.2, 28.5, 28.6, 28.8, 28.9, 29.2, 30.1, 46.6, 46.8, 47.0, 52.2, 52.3, 57.8, 57.9, 58.8, 79.6, 153.9, 154.7, 171.3, 171.7, 172.8, 173.1.
HRMS ESI-(+) calcd. for $C_{16}H_{26}N_2O_5Na$ $[M+Na]^+$ 349.1739, found 349,1737.

Cyclo(Pro-Pro) (9)

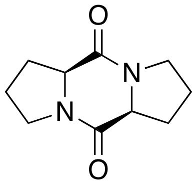

9

Procedure:
a) From Z-Pro-Pro-OMe (**7**): to a solution of the dipeptide **7** (300 mg, 0.83 mmol) in anhydrous MeOH (9 mL) was added 10% Pd/C (46 mg) and $NaHCO_3$ (77 mg, 0.92 mmol, 1.1 equiv). The reaction mixture was placed under hydrogen atmosphere and stirred at rt for 24 h. The reaction mixture was filtered on Celite, rinsed with MeOH, and the filtrate was concentrated under reduced pressure. The residue was taken up in dichloromethane, filtered and evaporated in vacuo to furnish the crude DKP as a slightly yellow solid. Purification by

flash chromatography on silica (dichloromethane/MeOH 100/0 to 95/05) allowed to obtain pure DKP **9** (135 mg, 0.69 mmol, 83%) as a white solid.

b) From Boc-Pro-Pro-OMe (**8**): the Boc-protected dipeptide **8** (90 mg, 0.27 mmol) was submitted to gaseous HCl for 2 h.[14] The crude hydrochloride (72 mg) was then dissolved in anhydrous MeOH (3 mL) and stirred for 24 h in the presence of an excess of $NaHCO_3$ (57 mg, 0.68 mmol, 2.5 equiv). The reaction mixture was concentrated under reduced pressure, the residue taken up in dichloromethane, filtered and the solvent removed under vacuum. The crude product was purified by flash chromatography on silica (dichloromethane/MeOH 100/0 to 95/05) to furnish pure DKP **9** (38 mg, 0.19 mmol, 70% over 2 steps) as a white solid.

Analytical data were in agreement with those reported in the literature.[15]

^{1}H NMR (400 MHz, $CDCl_3$) δ (ppm): 1.84-1.94 (m, 2H), 1.96-2.06 (m, 2H), 2.12-2.22 (m, 2H), 2.27-2.35 (m, 2H), 3.52 (d, 2H, *J* = 8.6 Hz), 3.53 (dd, 2H, *J* = 8.6, 1.6 Hz), 4.16 (t, 2H, *J* = 8.1 Hz).
^{13}C NMR (100 MHz, CDCl3) δ (ppm): 23.4, 27.8, 45.3, 60.6, 166.5.
HRMS ESI-(+) calcd. for $C_{10}H_{15}N_2O_2$ $[M+H]^+$ 195.1134, found 195.1136.

meso-Dimethyl-2,5-dibromohexanedioate (meso-**10**)

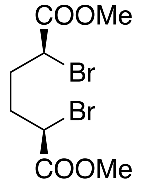

meso-10

Procedure: thionyl chloride (200 g, 1.68 mol, 2.45 equiv) was added dropwise to adipic acid (100 g, 0.685 mol) under stirring. At the end of the addition, the reaction mixture was heated at 70–80 °C for 3 h. After cooling to rt, the excess of thionyl chloride was removed by evaporation under reduced pressure. Bromine (250 g, 1.56 mol, 2.27 equiv) was then added dropwise to the resulting acid chloride over 6 h at 80 °C. After the addition, the reaction mixture was stirred for 12 h at rt, then poured in a dropping funnel and added slowly to 500 mL of anhydrous methanol at −5 °C. The solution was stirred at rt for 12 h, after which meso dimethyl dibromoadipate (**10**) precipitates by cooling of the methanolic solution. The resulting solid was filtered, washed several times with cold methanol and recrystallized from methanol. The filtrate was evaporated under vacuum, the oily residue dissolved in diethyl ether (500 mL) and washed successively with 2% aqueous sodium bisulfite solution (100 mL), 3% aqueous sodium hydrogenocarbonate solution (100 mL) and water. The organic phase was dried with K_2CO_3 (17 g) and concentrated. The oily residue was dissolved in a minimal amount of methanol and allowed to stand at 5–7 °C until precipitation of residual meso compound. The crystallized product was filtered, washed and recrystallized from methanol. The gathered solids gave 183 g (0,55 mol, 83%) of the title compound meso-**10**.[16]

^{1}H NMR (300 MHz, $CDCl_3$) δ (ppm): 2.02-2.12 (m, 2H), 2.23-2.37 (m, 2H), 3.80 (s, 6H), 4.23-4.28 (m, 2H).
^{13}C NMR (100 MHz, CDCl3) δ (ppm): 32.6, 44.4, 53.3, 169.8.
m.p.: 76 °C (76-77 °C litt.)

cis-2,5-Dimethyl-*N*-benzylpyrrolidine-2,5-dicarboxylate (*cis*-**11**) and *trans*-2,5-dimethyl-*N*-benzylpyrrolidine-2,5-dicarboxylate (*trans*-**11**)

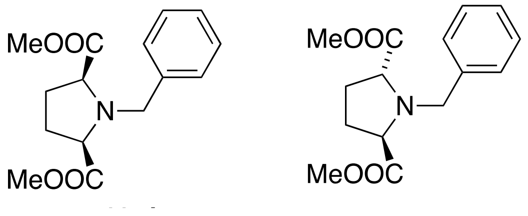

cis-**11** *trans*-**11**

Procedure: Dimethyl (*R*,*S*)-2,5-dibromohexanedioate **10** (785 mg, 2.36 mmol, 1 equiv), benzylamine (340 µL, 3.10 mmol, 1.3 equiv) and potassium carbonate (720 mg, 5.21 mmol, 2.2 equiv) and EtOAc (600 µL, η = 0.33 µL.mg^{-1}) as liquid grinding assistant were introduced in a 45 mL stainless steel grinding bowl with 19 stainless steel balls (10 mm diameter). The bowl was closed and placed for 120 min within the planetary mill at 500 rpm. Then, the reaction mixture was taken up in EtOAc, filtered and washed with water and brine. After drying on $MgSO_4$, the organic layer was evaporated under vacuum to furnish the crude product as a colourless oil (*cis*/*trans* ratio of 97/03 measured by ^{1}H NMR). Purification by flash chromatography on silica (cyclohexane/EtOAc 100/0 to 80/20) allowed to isolate the expected pyrrolidine *cis*-**11** (490 mg, 1.77 mmol, 75% yield) and the minor isomer *trans*-**11** (15 mg, 0.05 mmol, 2.3%), each as colourless oil. Analytical data were in agreement with those reported in the literature for both isomers.[17]

cis-2,5-Dimethyl-*N*-benzylpyrrolidine-2,5-dicarboxylate (*cis*-**11**)

TLC: Rf = 0.35 (cyclohexane/EtOAc 80/20)
^{1}H NMR (300 MHz, $CDCl_3$) δ (ppm): 2.06-2.08 (m, 4H), 3.44-3.49 (m, 2H), 3.58 (s, 6H), 3.94 (s, 2H), 7.21-7.34 (m, 5H).
^{13}C NMR (100 MHz, CDCl3) δ (ppm): 28.7, 52.0, 57.8, 65.5, 127.5, 128.2, 129.8, 137.0, 173.8.
HRMS ESI-(+) calcd. for $C_{15}H_{20}NO_4$ $[M+H]^+$ 278.1392, found 278.1395.

trans-2,5-Dimethyl-*N*-benzylpyrrolidine-2,5-dicarboxylate (*trans*-**11**)

TLC: Rf = 0.53 (cyclohexane/EtOAc 80/20)
Appearance: slight yellow oil
^{1}H NMR (400 MHz, $CDCl_3$) δ (ppm): 1.89-1.97 (m, 2H), 2.26-2.37 (m, 2H), 3.64 (s, 6H), 3.79 (d, 1H, *J* = 13,0 Hz), 3.82-3.86 (m, 2H), 3.98 (d, *J* = 13.0 Hz, 1H), 7.21-7.33 (m, 5H).
^{13}C NMR (100 MHz, CDCl3) δ (ppm): 28.5, 51.7, 54.3, 63.5127.4, 128.4, 129.2, 138.4, 174.6.
HRMS ESI-(+) calcd. for $C_{15}H_{20}NO_4$ $[M+H]^+$ 278.1392, found 278.1394.

cis-Dimethyl-2,5-pyrrolidine-2,5-dicarboxylate (**12**)

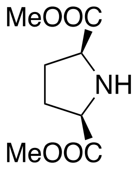

12

Procedure: To a solution of pyrrolidine *cis*-**11** (280 mg, 1.01 mmol) in methanol (5 mL) was added 10% Pd/C (55 mg). The reaction mixture was placed under a hydrogen atmosphere and stirred overnight at rt The mixture was filtered on Celite, rinsed with methanol, and the filtrate was evaporated under vacuum. The debenzylated pyrrolidine **12** (183 mg, 0.96 mmol, 95%) was obtained as a yellow oil and used without further purification. Analytical data obtained were in agreement with those reported in the literature.[18]

^{1}H NMR (400 MHz, CDCl$_3$) δ (ppm): 1.89-1.97 (m, 2H), 2.10-2.17 (m, 2H), 2.63 (br s, 1H), 3.73 (s, 6H), 3.79-3,84 (m, 2H).
^{13}C NMR (100 MHz, CDCl3) δ (ppm): 29.9, 52.4, 60.4, 174.7.
HRMS ESI-(+) calcd. for $C_8H_{14}NO_4$ $[M+H]^+$ 188.0925, found 188.0923.

Z-Pro-Pyr-(OMe)$_2$ **(13)**

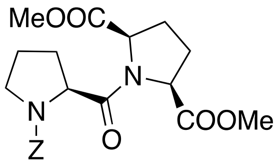

13

Procedure: *cis*-Pyrrolidine **12** (130 mg, 0.69 mmol), (*S*)-*N*-benzyloxycarbonylproline 210 mg, 0.83 mmol, 1.2 equiv) **5**, NaH_2PO_4 (250 mg, 2.10 mmol, 3 equiv), oxyma (120 mg, 0.83 mmol, 1.2 equiv), EDCI (185 μL, 1.04 mmol, 1.5 equiv) and EtOAc (400 μL, η = 0.46 μL.mg^{-1}) as liquid assistant were introduced in a 10 mL stainless steel grinding jar with one stainless steel ball (10 mm diameter). The jar was closed and submitted to vibration milling for 1 h at 30 Hz. The residue was then dissolved in EtOAc and water, the layers were separated and the organic one was washed with 1 N aqueous HCl solution, saturated aqueous $NaHCO_3$ solution and brine. After drying over $MgSO_4$, filtration and evaporation under vacuum, the pseudo-dipeptide **13** (234 mg, 0.54 mmol, 78%) was obtained as a slight yellow oil and engaged in the next step without further purification.

TLC: Rf = 0.26 (cyclohexane/EtOAc 50/50) (UV, ninhydrin)
^{1}H NMR (600 MHz, CDCl$_3$) δ (ppm): (rotamers) 1.77-1.89 (m, 2H), 1.93-2.39 (m, 6H), 3.40-3.67 (m, 2H), 3.68 (s, 1.5H), 3.72 (s, 1.5H), 3.73 (s, 1.5H), 3.78 (s, 1.5H), 4.26-4.33 (m, 1.2H), 4.46 (dd, 0.6H, *J* = 7.9, 4.5 Hz), 4.61 (t, 0.6H, *J* = 15.8 Hz), 4.88 (d, 0.4H, *J* = 11.7 Hz), 5.02 (d, 0.6H, *J* = 12.5 Hz), 5.11-5.16 (m, 1.6H), 7.28-7.38 (m, 5H).

^{13}C NMR (150 MHz, CDCl3) δ (ppm): (rotamers) 23.9, 24.8, 27.4, 27.8, 29.1, 29.8, 29.9, 30.1, 31.3, 47.2, 47.8, 52.3, 52.7, 57.3, 57.9, 59.7, 59.8, 59.9, 60.1, 67.1, 67.6, 127.7, 128.1, 128.6, 128.8, 128.9, 136.5, 136.7, 153.9, 155.2, 171.4, 171.6, 171.8, 171.9, 172.9, 173.0.
HRMS ESI-(+) calcd. for $C_{21}H_{27}N_2O_7$ $[M+H]^+$ 419.1818, found 419.1823.

Boc-Pro-Pyr-(OMe)$_2$ **(14)**

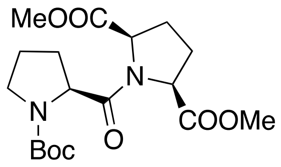

14

Procedure: cis-Pyrrolidine **12** (94 mg, 0.50 mmol), (*S*)-*N*-*tert*-butyloxycarbonylproline **6** (130 mg, 0.60 mmol, 1.2 equiv), NaH_2PO_4 (180 mg, 1.50 mmol, 3 equiv), oxyma (86 mg, 0.60 mmol, 1.2 equiv), EDCI (107 μL, 0.60 mmol, 1.2 equiv) and EtOAc (100 μL, η = 0.17 μL.mg^{-1}) as liquid assistant were introduced in a 15 mL PTFE grinding jar with one stainless steel ball (10 mm diameter). The jar was closed and submitted to vibration milling for 1 h at 30 Hz. The residue was then dissolved in EtOAc and water, the layers were separated and the aqueous layer was extracted once with EtOAc. The combined organic phases were washed with 1 N aqueous HCl solution, saturated aqueous $NaHCO_3$ solution and brine. After drying over $MgSO_4$, filtration and evaporation under vacuum, the pseudo-dipeptide **14** (141 mg, 0.36 mmol, 61%) was obtained as a colorless wax and engaged in the next step without further purification.

TLC: Rf = 0.27 (cyclohexane/EtOAc 50/50) (ninhydrin)
^{1}H NMR (600 MHz, $CDCl_3$) δ (ppm): (rotamers) 1.39, 1.41, 1.43 (s, 9H), 1.80 (m, 1H), 1.96-2.38 (m, 7H), 3.37 (m, 0.6H), 3.44 (m, 0.4H), 3.51 (m, 0.6H), 3.59 (m, 0.4H), 3.70, 3.72 (s, 3H), 3.76, 3.79 (s, 3H), 4.30 (dd, 0.3H, J = 7.7, 4.1 Hz), 4.40 (dd, 0.6H, *J* = 7.7, 4.1 Hz), 4.5-4.54 (m, 0.2H), 4.57 (m, 0.9H), 4.68 (dd, 0.3H, *J* = 8.2, 2.2 Hz), 5.10 (dd, 0.6H, *J* = 7.7, 2.7 Hz).
^{13}C NMR (150 MHz, CDCl3) δ (ppm): (rotamers) 23.6, 24.6, 27.6, 27.8, 28.6, 28.7, 30.0, 30.1, 30.3, 31.3, 47.3, 47.3, 52.2, 52.6, 52.8, 57.3, 57.5, 59.9, 60.0, 79.6, 79.8, 153.5, 154.7, 171.5, 171.5, 171.9, 173.1, 173.4.
HRMS ESI-(+) calcd. for $C_{18}H_{28}N_2O_7Na$ $[M+Na]^+$ 407.1794, found 407.1795.
m.p.: 122.5-123.5 °C.

<u>Diketopiperazine</u> **15a**

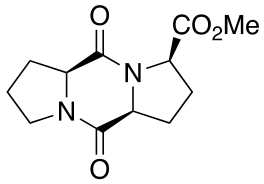

15a

Procedure:

<u>a) From Z-pseudo-dipeptide **13**:</u> to a solution of **13** (160 mg, 0.38 mmol) in anhydrous MeOH (5.0 mL) was added 10% Pd/C (25 mg) and $NaHCO_3$ (35 mg, 0.42 mmol, 1.1 equiv). The reaction mixture was placed under hydrogen atmosphere and stirred at rt for 20 h. The reaction mixture was filtered on Celite, rinsing with MeOH, and the filtrate was concentrated under reduced pressure. The residue was taken up in dichloromethane, filtered and evaporated *in vacuo* to furnish the expected DKP **15a** as a white solid (92 mg, 0.36 mmol, 95%).

<u>b) From Boc-pseudo-dipeptide **14**:</u> the Boc-protected pseudo-dipeptide **14** (140 mg, 0.36 mmol) was submitted to gaseous HCl for 2 h. The crude hydrochloride (116 mg) was then dissolved in anhydrous MeOH (4 mL) and stirred for 24 h in the presence of an excess of $NaHCO_3$ (76 mg, 0.90 mmol, 2.5 equiv). The reaction mixture was concentrated under reduced pressure, the residue taken up in dichloromethane, filtered and the solvent removed under vacuum. The crude product was purified by flash chromatography on silica (dichloromethane/MeOH 100/0 to 95/05) to furnish the expected DKP **15a** (73 mg, 0.29 mmol, 80% over 2 steps) as a white solid.

TLC: Rf = 0.55 (dichloromethane/MeOH 95/05) (potassium permanganate)
^{1}H NMR (400 MHz, $CDCl_3$) δ (ppm): 1.91 (m, 1H), 2.01 (m, 1H), 2.15-2.33 (m, 6H), 3.52 (ddd, 1H, *J* = 4.0, 8.3, 11.9 Hz), 3.64 (dt, 1H, J = 7.9, 11.5 Hz), 3.71 (s, 3H), 4.18 (t, 1H, *J* = 8.1 Hz), 4.28 (t, 1H, *J* = 7.8 Hz), 4.52 (m, 1H).
^{13}C NMR (100 MHz, CDCl3) δ (ppm): 23.5, 26.3, 27.7, 28.9, 45.4, 52.7, 58.0, 60.5, 61.2, 166.2, 166.9, 171.2.
HRMS ESI-(+) calcd. for $C_{12}H_{17}N_2O_4$ $[M+H]^+$ 253.1188, found 253.1191.
m.p.: 122.5-123.5 °C
RX:

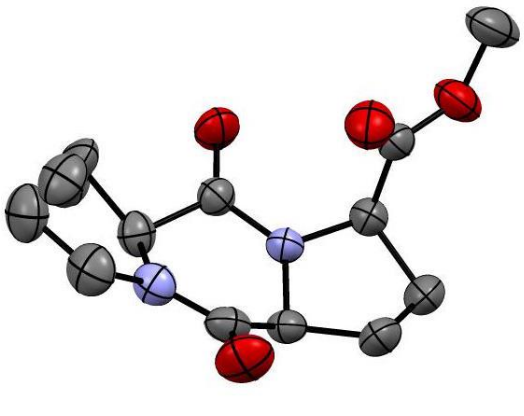

(2*S*,5*R*)-*N*-benzyl-5-methoxycarbonylproline **17**

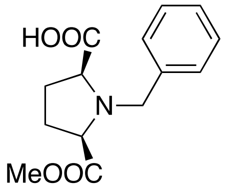

17

Procedure: diester **11*cis*** (135 mg, 0.48 mmol) was dissolved in acetone (1.50 mL) and 0,06 M phosphate buffer of pH 8 (12.5 mL). PLE (15 mg, 270 units) was added and the emulsion stirred vigorously at 30 °C for 4 h. The reaction mixture was acidified with HCl 1 N to pH 1–2, concentrated under reduced pressure and extracted several times with EtOAc. The combined organic layers were dried over $MgSO_4$, filtered and evaporated under vacuum to furnish the half ester **17** as a viscous yellow oil (100 mg, 0.38 mmol, 79%). Analytical data were in agreement with those reported in the literature.[19]

^{1}H NMR (400 MHz, $CDCl_3$) δ (ppm): 1.89 (m, 1H), 2.14 (m, 1H), 2.20-2.35 (m, 2H), 3.65 (s, 3H), 3.73-3,84 (m, 2H), 3.97 (s, 2H), 7.26-7.33 (m, 5H).

Pseudodipeptide **18**

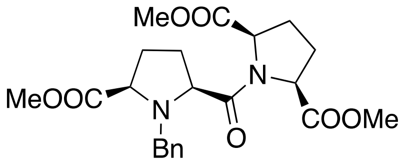

18

Procedure: acid **17** (80 mg, 0.30 mmol), *cis*-pyrrolidine **11** (70 mg, 0.37 mmol, 1.2 equiv), NaH_2PO_4 (145 mg, 1.20 mmol, 4 equiv), oxyma (88 mg, 0.60 mmol, 2 equiv), EDCI (110 μL, 0.60 mmol, 2 equiv) and EtOAc (50 μL, η = 0.46 μL.mg^{-1}) as liquid grinding assistant were introduced in a 15 mL PTFE grinding jar with one stainless steel ball (10 mm diameter). The jar was closed and subjected to grinding for 50 min in the vibratory ball mill operated at 30 Hz. The residue was then taken up in EtOAc, washed with 1 N aqueous HCl solution, saturated aqueous $NaHCO_3$ solution and brine. After drying over $MgSO_4$, filtration and evaporation under vacuum, the pseudo-dipeptide **18** (120 mg, 0.27 mmol, 90%) was obtained as a yellow wax and engaged in the next step without further purification.

Diketopiperazine **19**

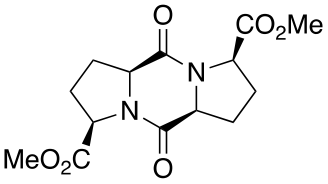

19

Procedure: to a solution of compound **18** (20 mg, 0.046 mmol) in anhydrous MeOH (2,0 mL) was added 20% $Pd(OH)_2/C$ (5 mg) and acetic acid (25 µL). The reaction mixture was placed under a hydrogen atmosphere and stirred at rt for 20 h. The reaction mixture was filtered with Celite, rinsed with MeOH, and the filtrate was concentrated under reduced pressure. The crude residue was purified by flash chromatography on silica (dichloromethane/MeOH 100/0 to 95/05) to furnish the expected DKP **19** (7,5 mg, 0.024 mmol, 52%) as a white solid.

TLC: Rf = 0.38 (dichloromethane/MeOH 95/05) (potassium permanganate)
^{1}H NMR (500 MHz, $CDCl_3$) δ (ppm): 2.18-2.34 (m, 8H), 3.75 (s, 6H), 4.32 (app. t, 2H, *J* = 8.1 Hz), 4.55 (app. d, 2H, *J* = 7.6 Hz).
^{13}C NMR (125 MHz, CDCl3) δ (ppm): 26.1, 29.1, 52.7, 58.1, 61.1, 166.6, 171.0.
HRMS ESI-(+) calcd. for $C_{14}H_{19}N_2O_6$ $[M+H]^+$ 311.1243, found 311.1243.
RX:

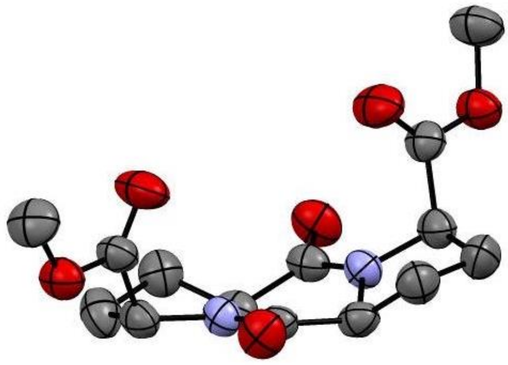

References

(1) Gaussian 09, Revision D01, M. J. Frisch, G. W. Trucks, H. B. Schlegel, G. E. Scuseria, M. A. Robb, J. R. Cheeseman, G. Scalmani, V. Barone, B. Mennucci, G. A. Petersson, H. Nakatsuji, M. Caricato, X. Li, H. P. Hratchian, A. F. Izmaylov, J. Bloino, G. Zheng, J. L. Sonnenberg, M. Hada, M. Ehara, K. Toyota, R. Fukuda, J. Hasegawa, M. Ishida, T. Nakajima, Y. Honda, O. Kitao, H. Nakai, T. Vreven, J. A. Montgomery, Jr., J. E. Peralta, F. Ogliaro, M. Bearpark, J. J. Heyd, E. Brothers, K. N. Kudin, V. N. Staroverov, R. Kobayashi, J. Normand, K. Raghavachari, A. Rendell, J. C. Burant, S. S. Iyengar, J. Tomasi, M. Cossi, N. Rega, J. M. Millam, M. Klene, J. E. Knox, J. B. Cross, V. Bakken, C. Adamo, J. Jaramillo, R. Gomperts, R. E. Stratmann, O. Yazyev, A. J. Austin, R. Cammi, C. Pomelli, J. W. Ochterski, R. L. Martin, K. Morokuma, V. G. Zakrzewski, G. A. Voth, P. Salvador, J. J. Dannenberg, S. Dapprich, A. D. Daniels, Ö. Farkas, J. B. Foresman, J. V. Ortiz, J. Cioslowski, and D. J. Fox, *Gaussian, Inc., Wallingford CT* **2009**.

(2) Chai, J.-D.; Head-Gordon, M. *Phys. Chem. Chem. Phys.* **2008**, *10*, 6615.

(3) Marenich, A. V.; Cramer, C. J.; Truhlar, D. G. *J. Phys. Chem. B* **2009**, *113*, 6378.

(4) Grimme, S.; Antony, J.; Ehrlich, S.; Krieg, H. *J. Chem. Phys.* **2010**, *132*, 154104.

(5) Grimme, S.; Ehrlich, S.; Goerigk, L. *J. Comput. Chem.* **2011**, *32*, 1456.

(6) Schäfer, A.; Horn, H.; Ahlrichs, R. *J. Chem. Phys.* **1992**, *97*, 2571.

(7) Weigend, F.; Ahlrichs, R. *Phys. Chem. Chem. Phys.* **2005**, *7*, 3297.

(8) Bonnamour, J.; Metro, T.-X.; Martinez, J.; Lamaty, F. *Green Chem.* **2013**, *15*, 1116.

(9) Bhuyan, B. J.; Mugesh, G. *Org. Biomol. Chem.* **2012**, *10*, 2237.

(10) Nayani, K.; Anwar Hussaini, S. D. *Tetrahedron Lett.* **2017**, *58*, 1166.

(11) Porte, V.; Thioloy, M.; Pigoux, T.; Métro, T.-X.; Martinez, J.; Lamaty, F. *Eur. J. Org. Chem.* **2016**, *2016*, 3505.

(12) Lu, D.; Gong, Y.; Wang, W. *Adv. Synth. Catal.* **2010**, *352*, 644.

(13) Gauchot, V.; Schmitzer, A. R. *J. Org. Chem.* **2014**, *79*, 2694.

(14) Declerck, V.; Nun, P.; Martinez, J.; Lamaty, F. *Angew. Chem. Int. Ed.* **2009**, *48*, 9318.

(15) Gautschi, M.; Schmid, J. P.; Peppard, T. L.; Ryan, T. P.; Tuorto, R. M.; Yang, X. *J. Agric. Food Chem.* **1997**, *45*, 3183.

(16) Lenda, F.; Guenoun, F.; Tazi, B.; Ben larbi, N.; Allouchi, H.; Martinez, J.; Lamaty, F. d. r. *Eur. J. Org. Chem.* **2005**, *2005*, 326.

(17) Bartuschat, A. L.; Wicht, K.; Heinrich, M. R. *Angew. Chem. Int. Ed.* **2015**, *54*, 10294.

(18) Sunilkumar, G.; Nagamani, D.; Argade, N. P.; Ganesh, K. N. *Synthesis* **2003**, 2304.

(19) Boutelje, J.; Hjalmarsson, M.; Hult, K.; Lindbaeck, M.; Norin, T. *Bioorg. Chem.* **1988**, *16*, 364.

NMR spectra of synthesized compounds

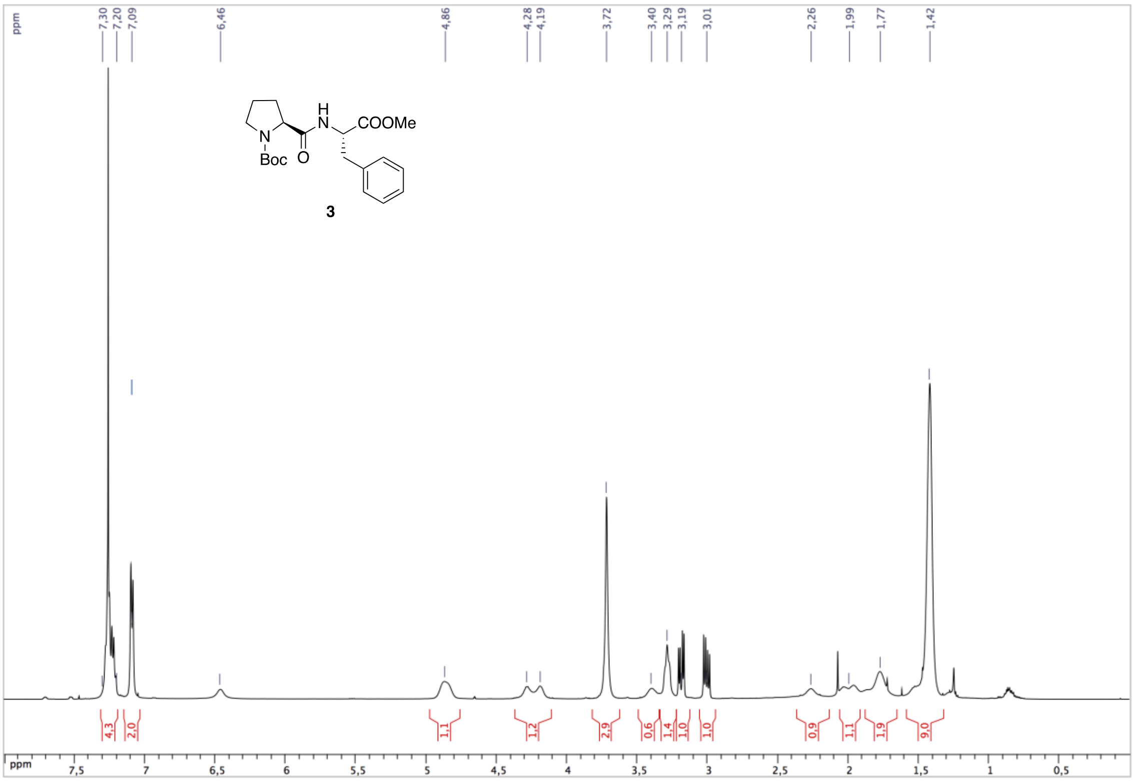

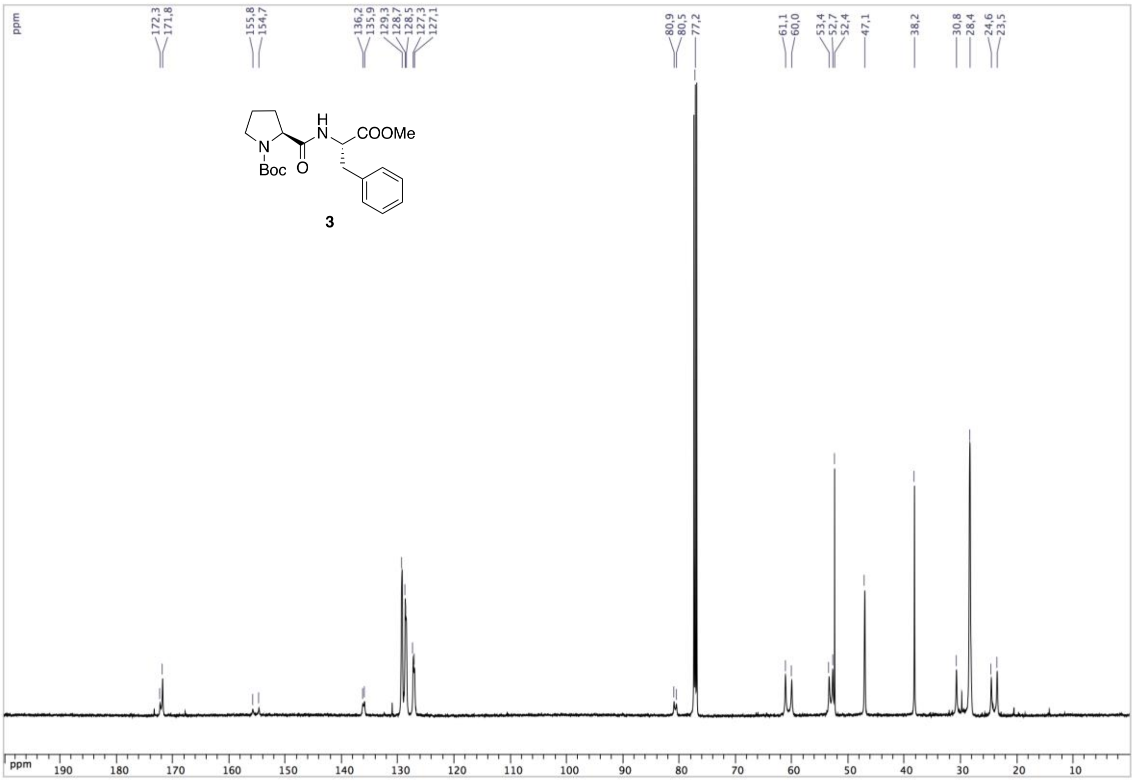

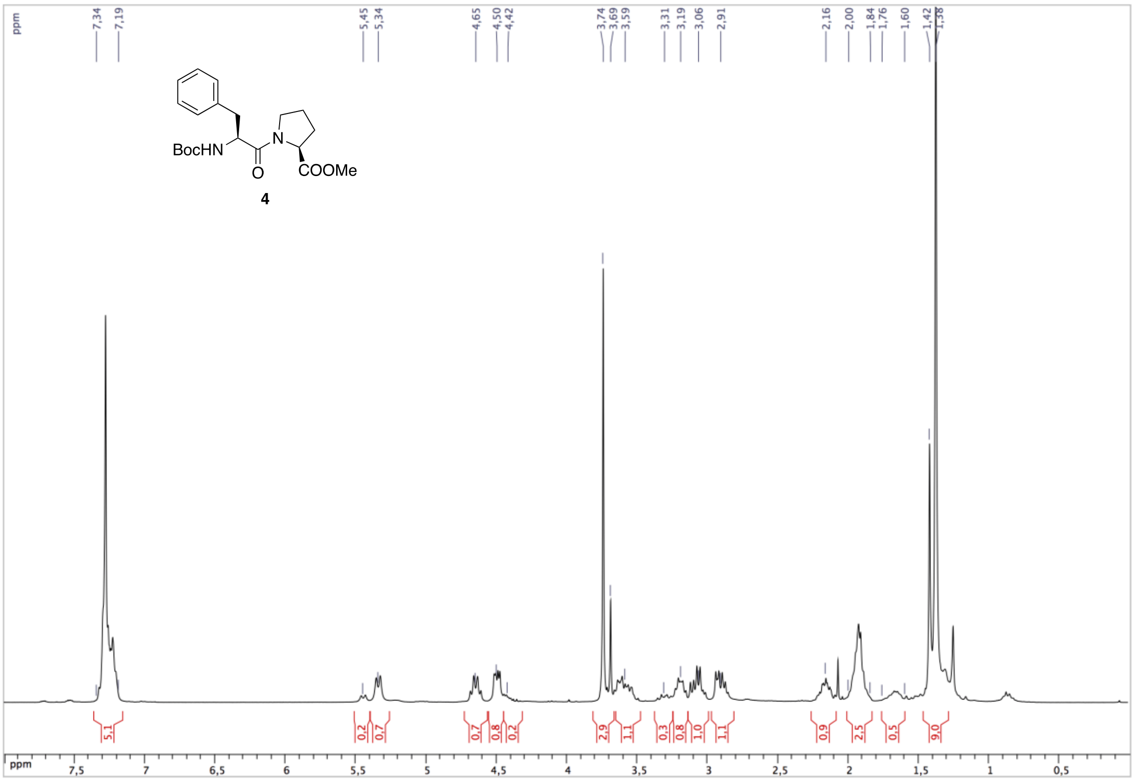
ppm
7,34
7,19
5,45
5,34
4,65
4,50
4,42
3,74
3,69
3,59
3,31
3,19
3,06
2,91
2,16
2,00
1,84
1,76
1,60
1,42
1,38
BocHN
O
N
COOMe
4
5,1
0,2
0,7
0,7
0,8
0,2
2,9
1,1
0,3
0,8
1,0
1,1
0,9
2,5
0,5
9,0
ppm
7,5
7
6,5
6
5,5
5
4,5
4
3,5
3
2,5
2
1,5
1
0,5

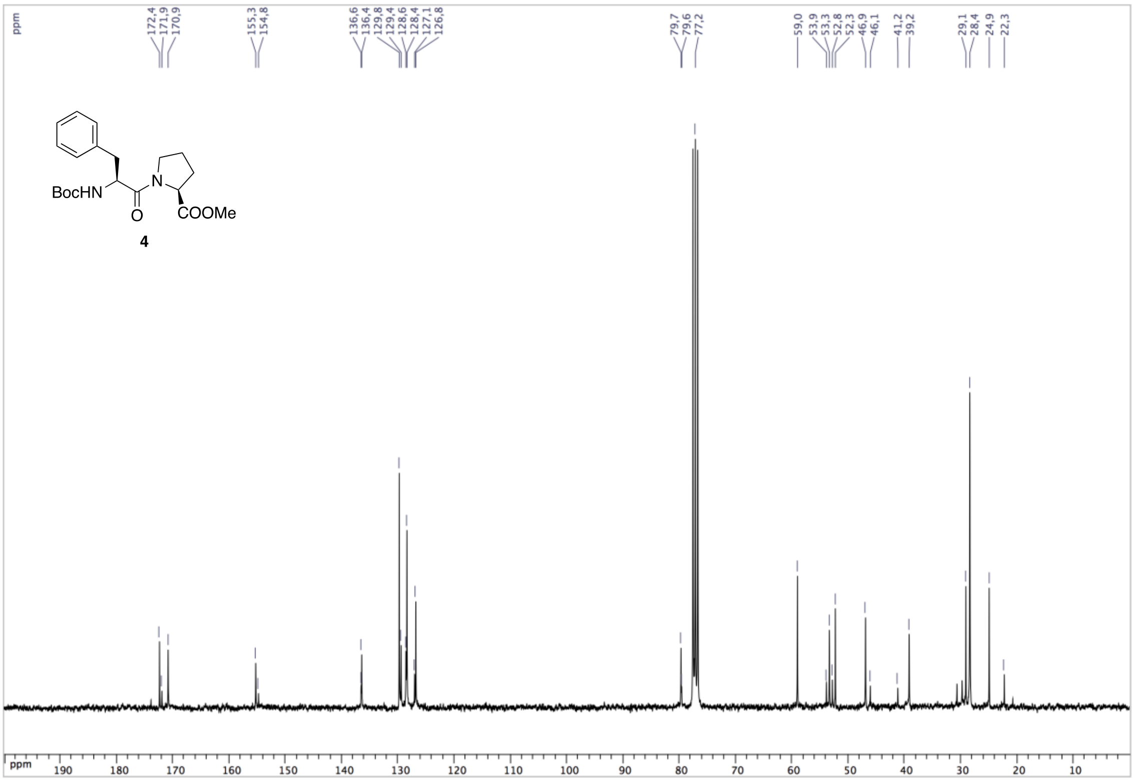
ppm
172,4
171,9
170,9
155,3
154,8
136,6
136,4
129,8
129,4
128,6
128,4
127,1
126,8
79,7
79,6
77,2
59,0
53,9
53,3
52,8
52,3
46,9
46,1
41,2
39,2
29,1
28,4
24,9
22,3
BocHN
O
N
COOMe
4
ppm
190
180
170
160
150
140
130
120
110
100
90
80
70
60
50
40
30
20
10

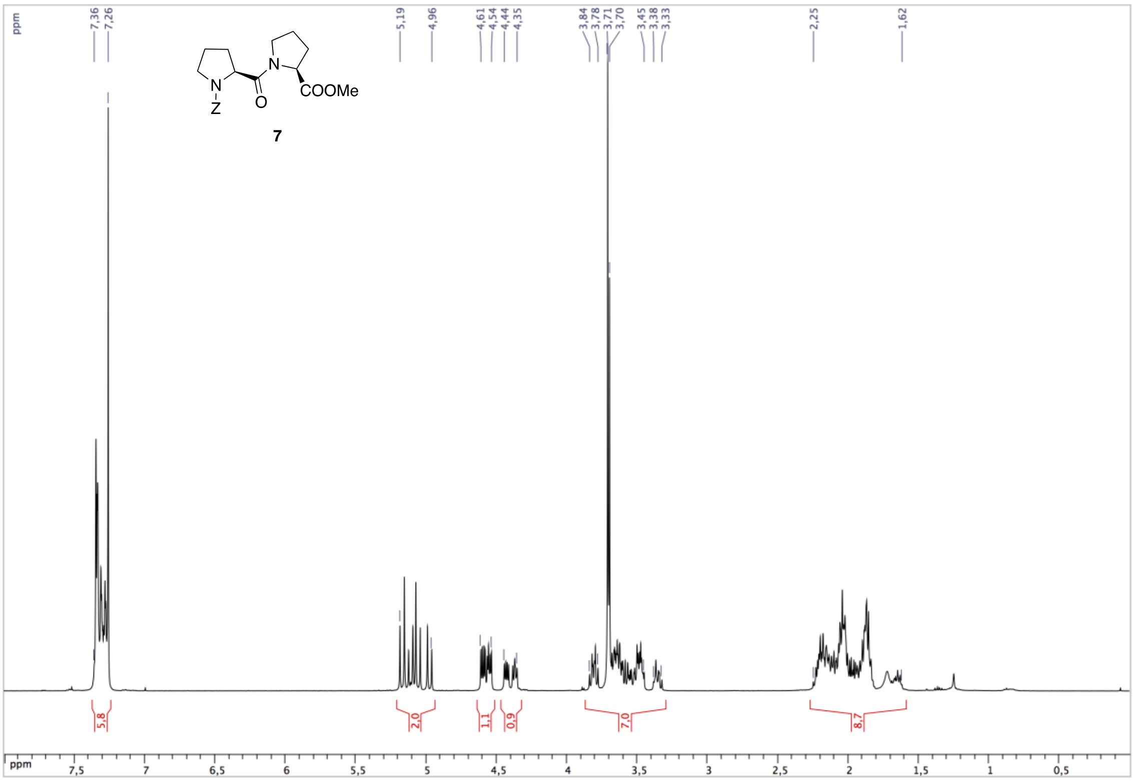
ppm
7,36
7,26
5,19
4,96
4,61
4,54
4,44
4,35
3,84
3,78
3,71
3,70
3,45
3,38
3,33
2,25
1,62
COOMe
N
O
Z
7
5,8
2,0
1,1
0,9
7,0
8,7
ppm
7,5
7
6,5
6
5,5
5
4,5
4
3,5
3
2,5
2
1,5
1
0,5

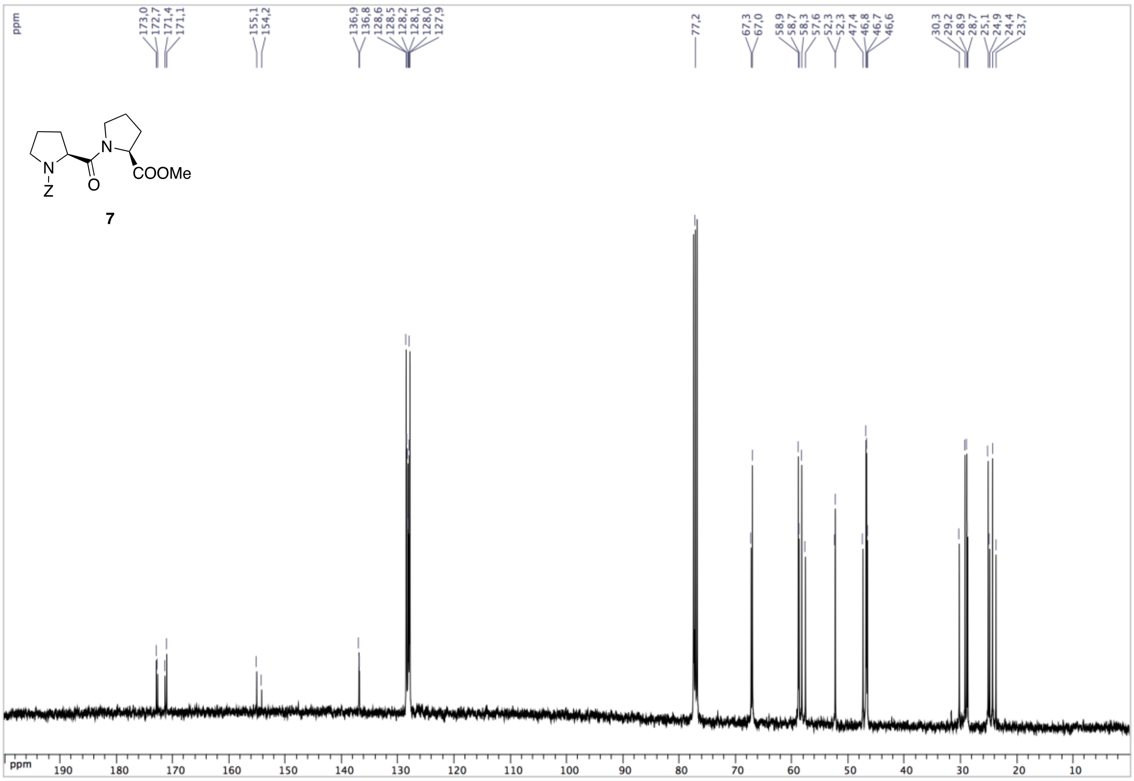
ppm
173,0
172,7
171,4
171,1
155,1
154,2
136,9
136,8
128,6
128,5
128,2
128,1
128,0
127,9
77,2
67,3
67,0
58,9
58,7
58,3
57,6
52,3
52,3
47,4
46,8
46,7
46,6
30,3
29,2
28,9
28,7
25,1
24,9
24,4
23,7
COOMe
N
O
Z
7
ppm
190
180
170
160
150
140
130
120
110
100
90
80
70
60
50
40
30
20
10

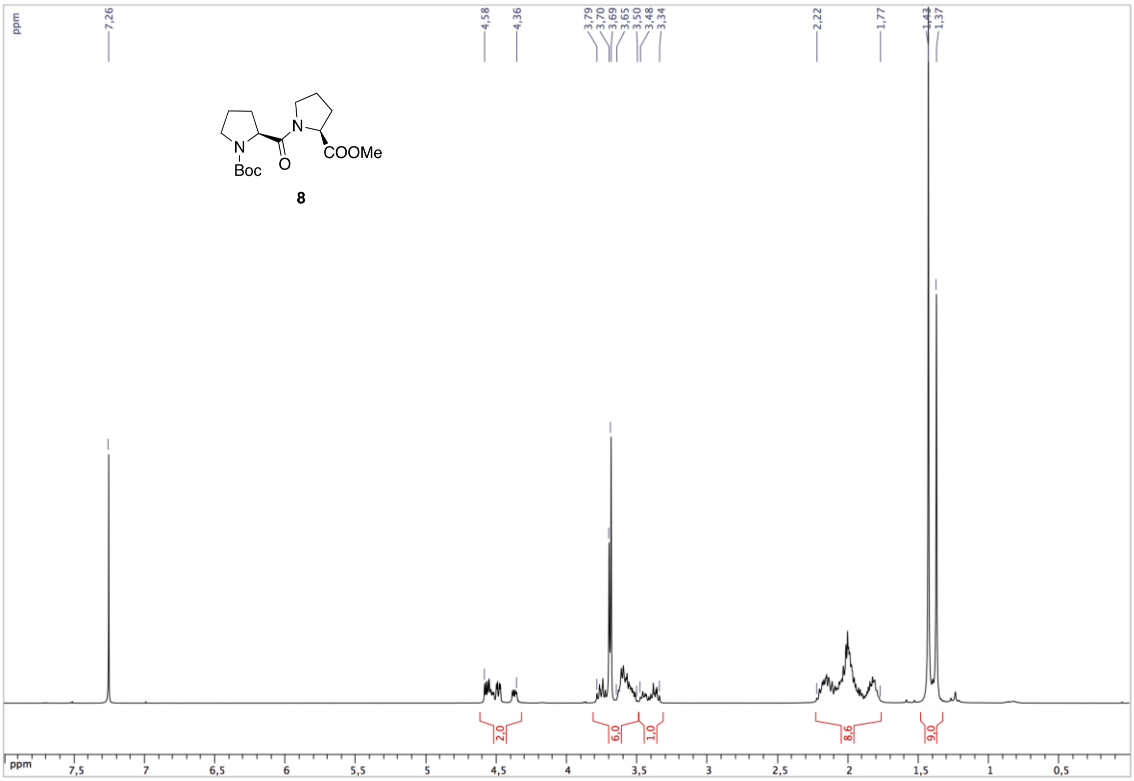
ppm
7,26
4,58
4,36
3,79
3,70
3,69
3,65
3,50
3,48
3,34
2,22
1,77
1,43
1,37
COOMe
Boc
8
2,0
6,0
1,0
8,6
9,0
ppm 7,5 7 6,5 6 5,5 5 4,5 4 3,5 3 2,5 2 1,5 1 0,5

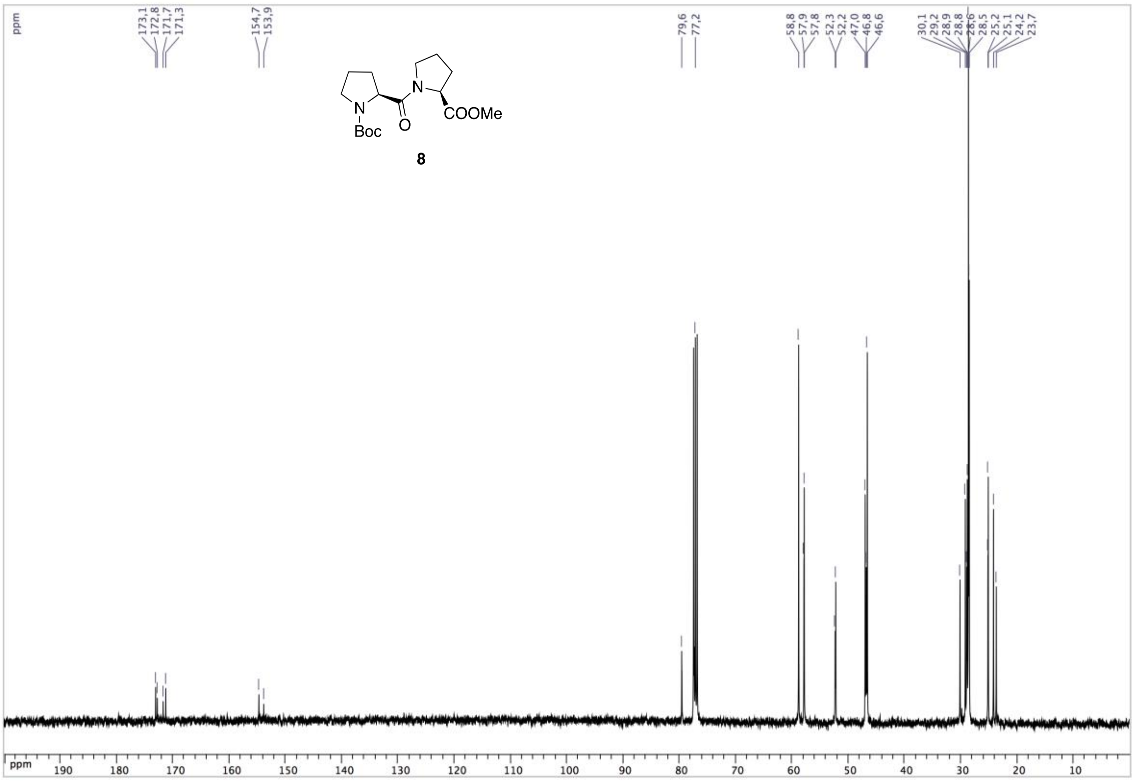
ppm
173,1
172,8
171,7
171,3
154,7
153,9
79,6
77,2
58,8
57,9
57,8
52,3
52,2
47,0
46,8
46,6
30,1
29,2
28,9
28,8
28,6
28,5
25,2
25,1
24,2
23,7
COOMe
Boc
8
ppm 190 180 170 160 150 140 130 120 110 100 90 80 70 60 50 40 30 20 10

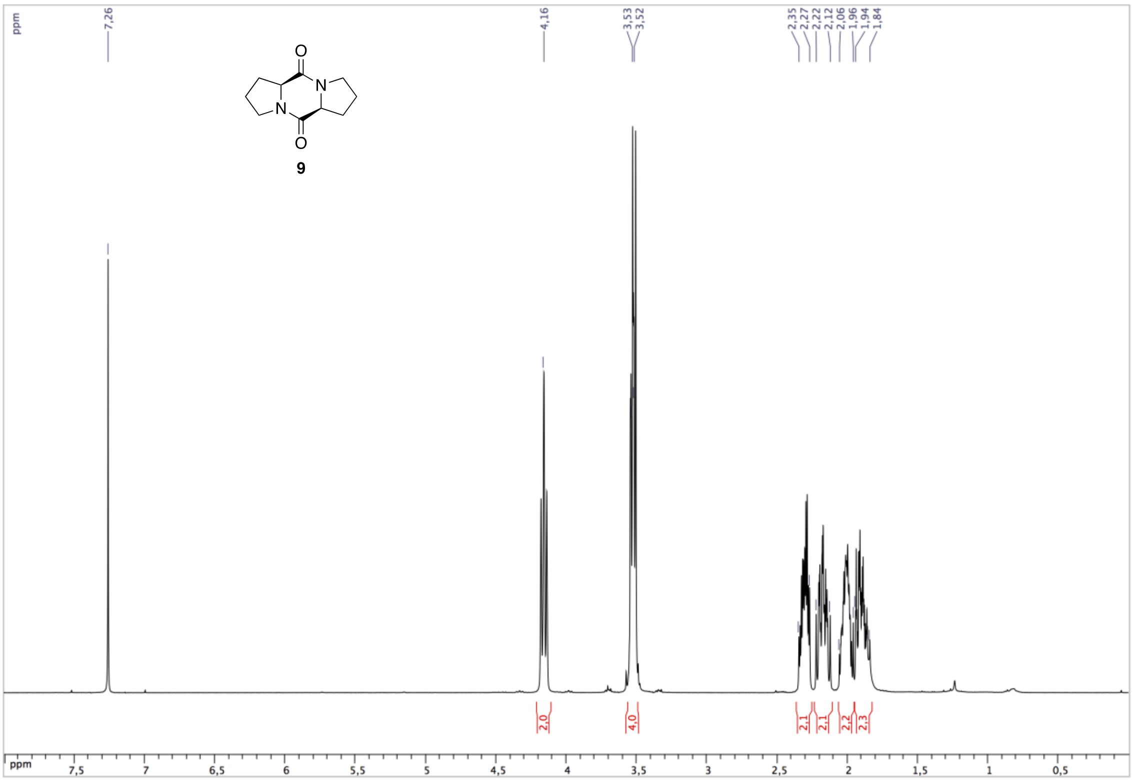
ppm
7,26
4,16
3,53
3,52
2,35
2,27
2,22
2,12
2,06
1,96
1,94
1,84
9
2,0
4,0
2,1
2,1
2,2
2,3
ppm 7,5 7 6,5 6 5,5 5 4,5 4 3,5 3 2,5 2 1,5 1 0,5

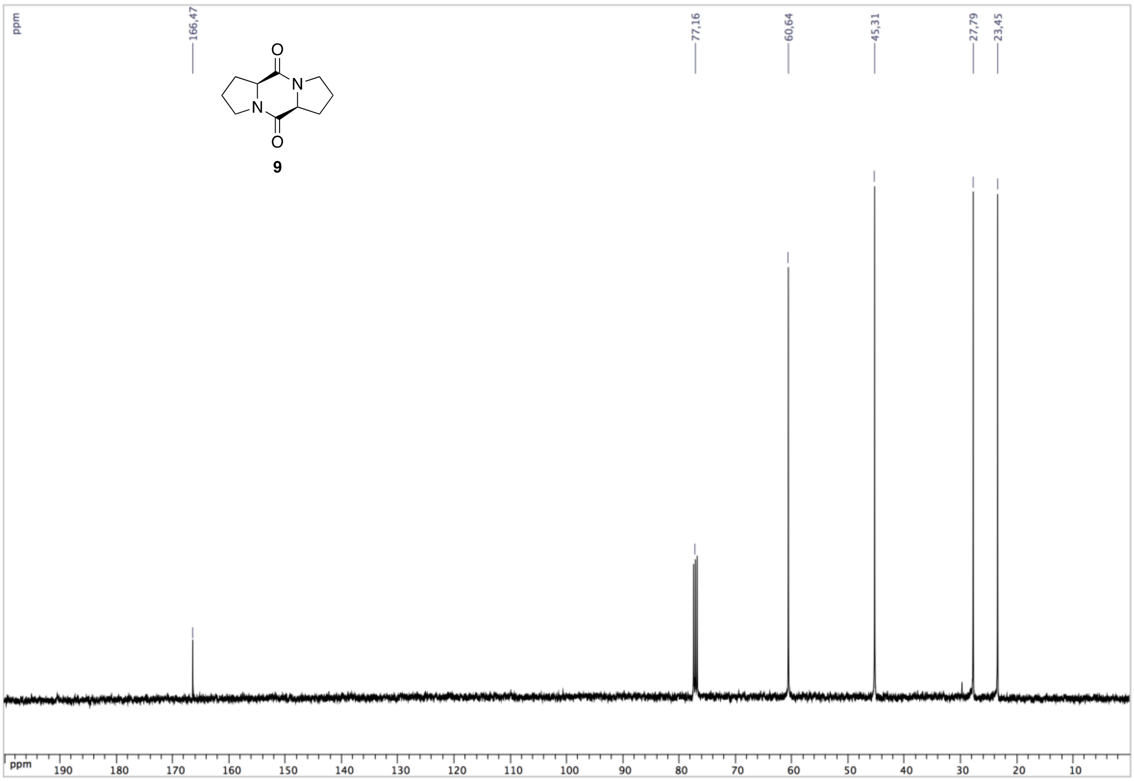
ppm
166,47
77,16
60,64
45,31
27,79
23,45
9
ppm 190 180 170 160 150 140 130 120 110 100 90 80 70 60 50 40 30 20 10

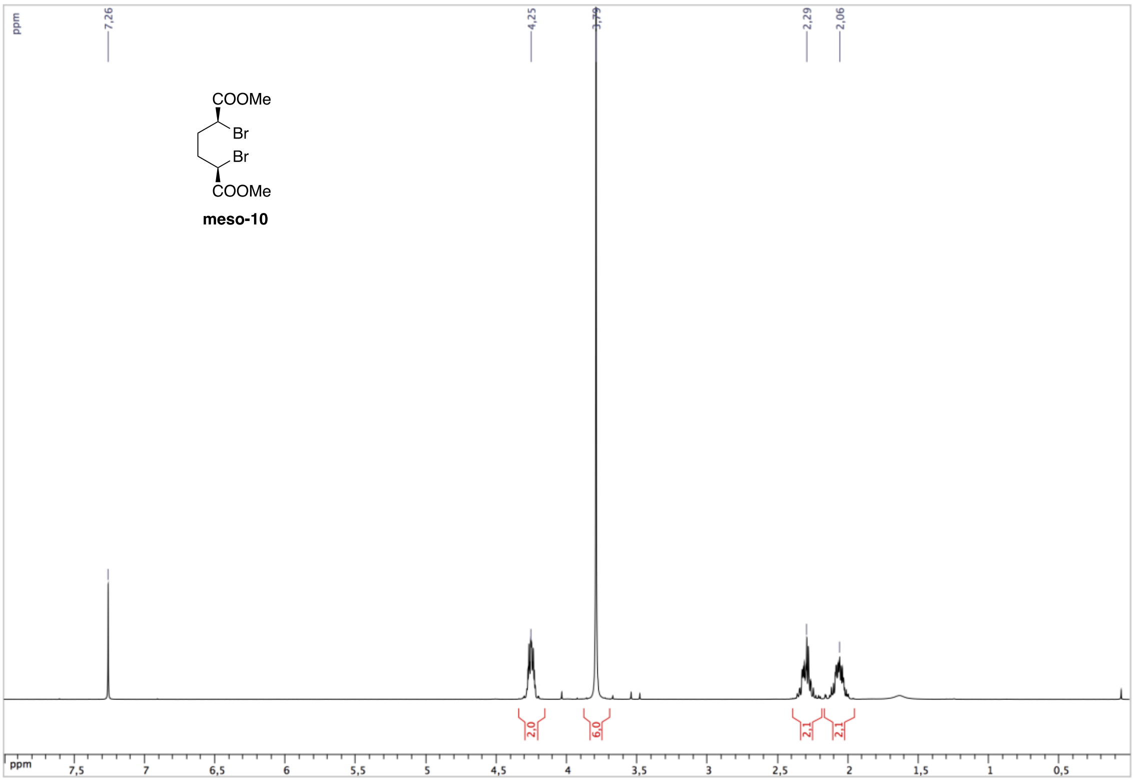
ppm
7,26
4,25
3,79
2,29
2,06
COOMe
Br
Br
COOMe
meso-10
2,0
6,0
2,1
2,1
ppm
7,5
7
6,5
6
5,5
5
4,5
4
3,5
3
2,5
2
1,5
1
0,5

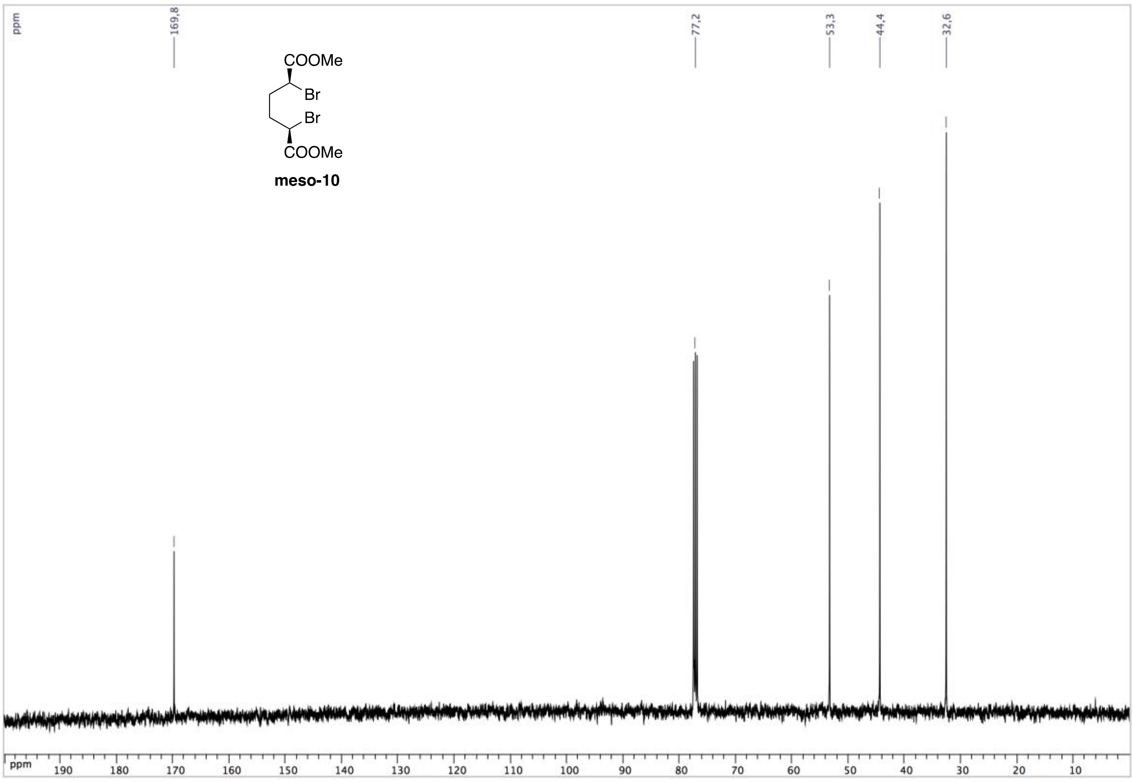
ppm
169,8
77,2
53,3
44,4
32,6
COOMe
Br
Br
COOMe
meso-10
ppm
190
180
170
160
150
140
130
120
110
100
90
80
70
60
50
40
30
20
10

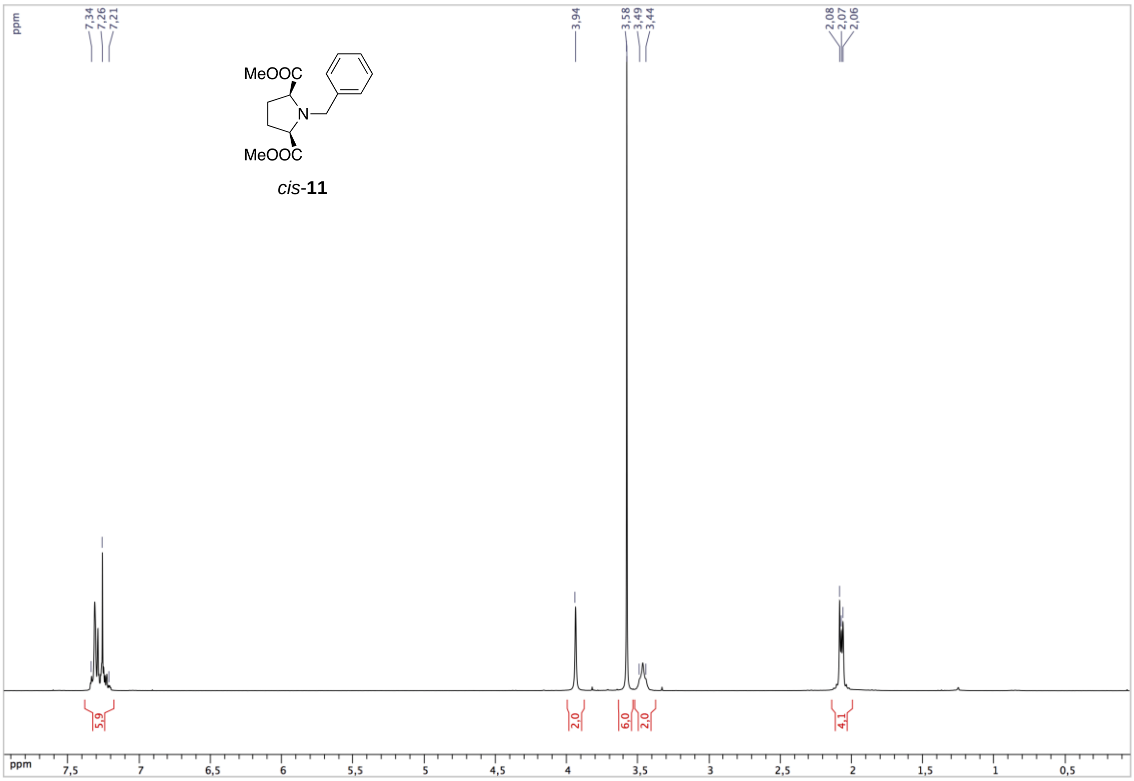
ppm
7,34
7,26
7,21
3,94
3,58
3,49
3,44
2,08
2,07
2,06
MeOOC
N
MeOOC
cis-11
5,9
2,0
6,0
2,0
4,1
ppm
7,5
7
6,5
6
5,5
5
4,5
4
3,5
3
2,5
2
1,5
1
0,5

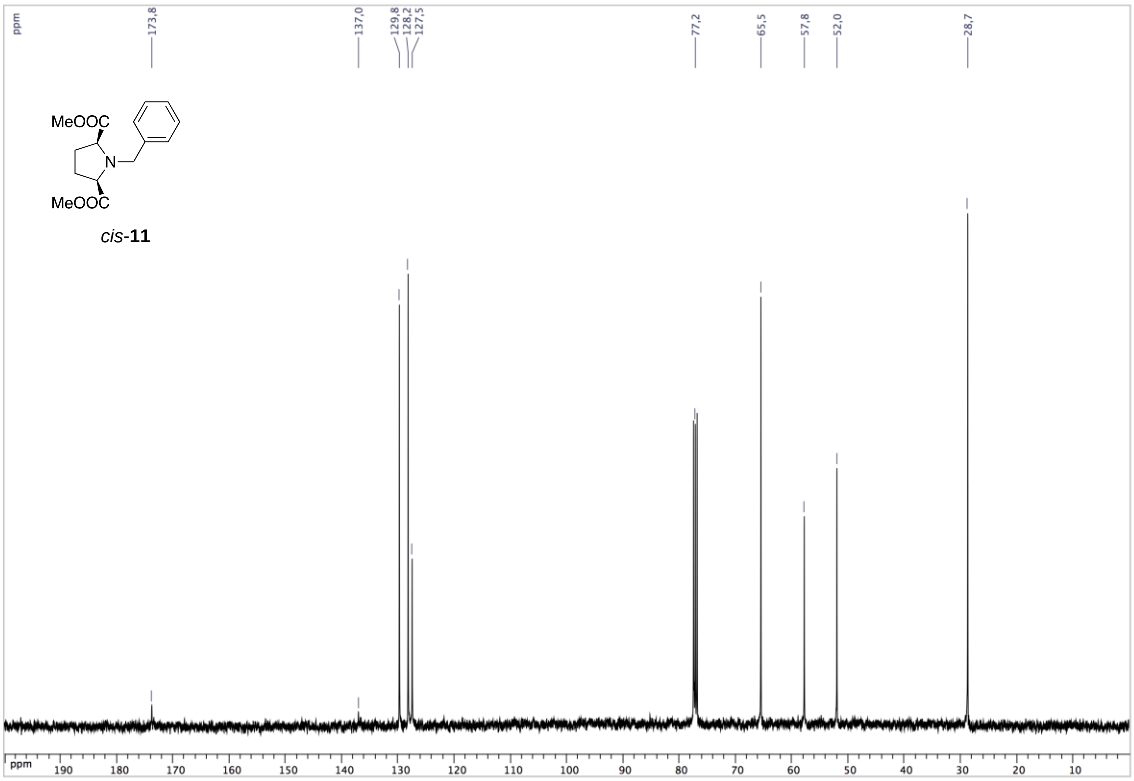
ppm
173,8
137,0
129,8
128,2
127,5
77,2
65,5
57,8
52,0
28,7
MeOOC
N
MeOOC
cis-11
ppm
190
180
170
160
150
140
130
120
110
100
90
80
70
60
50
40
30
20
10

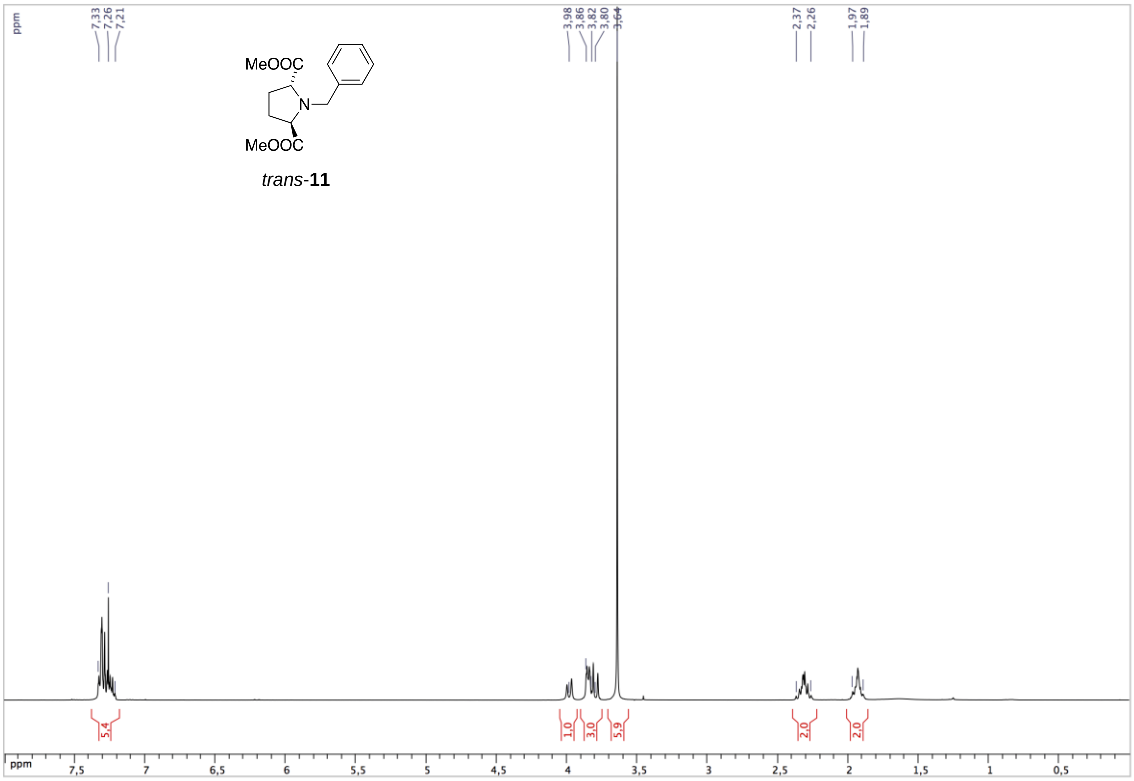
ppm
7,33
7,26
7,21
3,98
3,86
3,82
3,80
3,64
2,37
2,26
1,97
1,89
MeOOC
N
MeOOC
trans-11
5,4
1,0
3,0
5,9
2,0
2,0
ppm
7,5
7
6,5
6
5,5
5
4,5
4
3,5
3
2,5
2
1,5
1
0,5

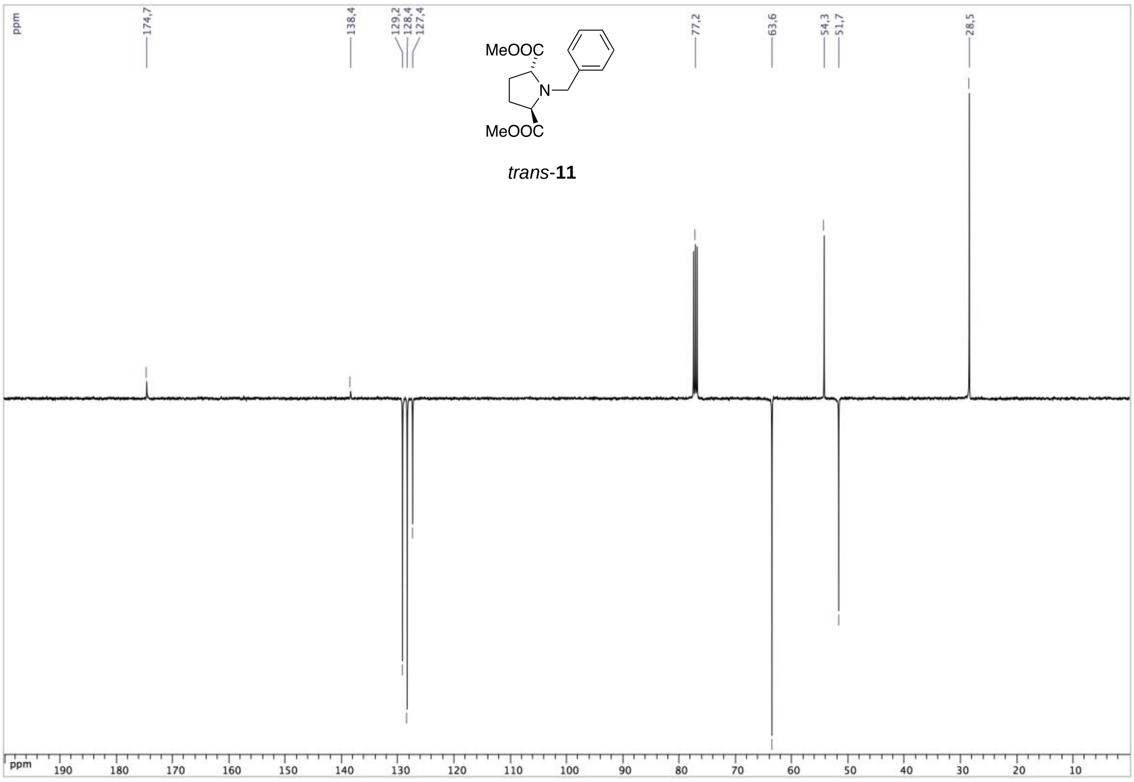
ppm
174,7
138,4
129,2
128,4
127,4
77,2
63,6
54,3
51,7
28,5
MeOOC
N
MeOOC
trans-11
ppm
190
180
170
160
150
140
130
120
110
100
90
80
70
60
50
40
30
20
10

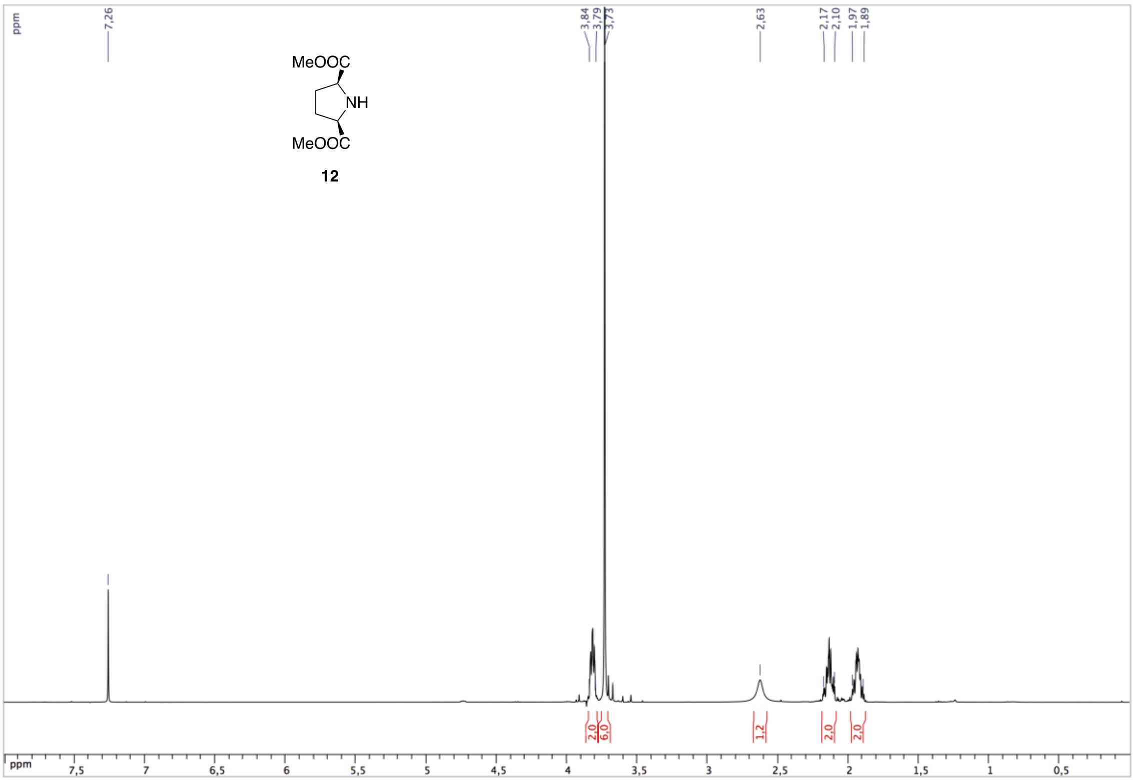
ppm
7,26
3,84
3,79
3,73
2,63
2,17
2,10
1,97
1,89
MeOOC
NH
MeOOC
12
2,0
6,0
1,2
2,0
2,0
ppm
7,5
7
6,5
6
5,5
5
4,5
4
3,5
3
2,5
2
1,5
1
0,5

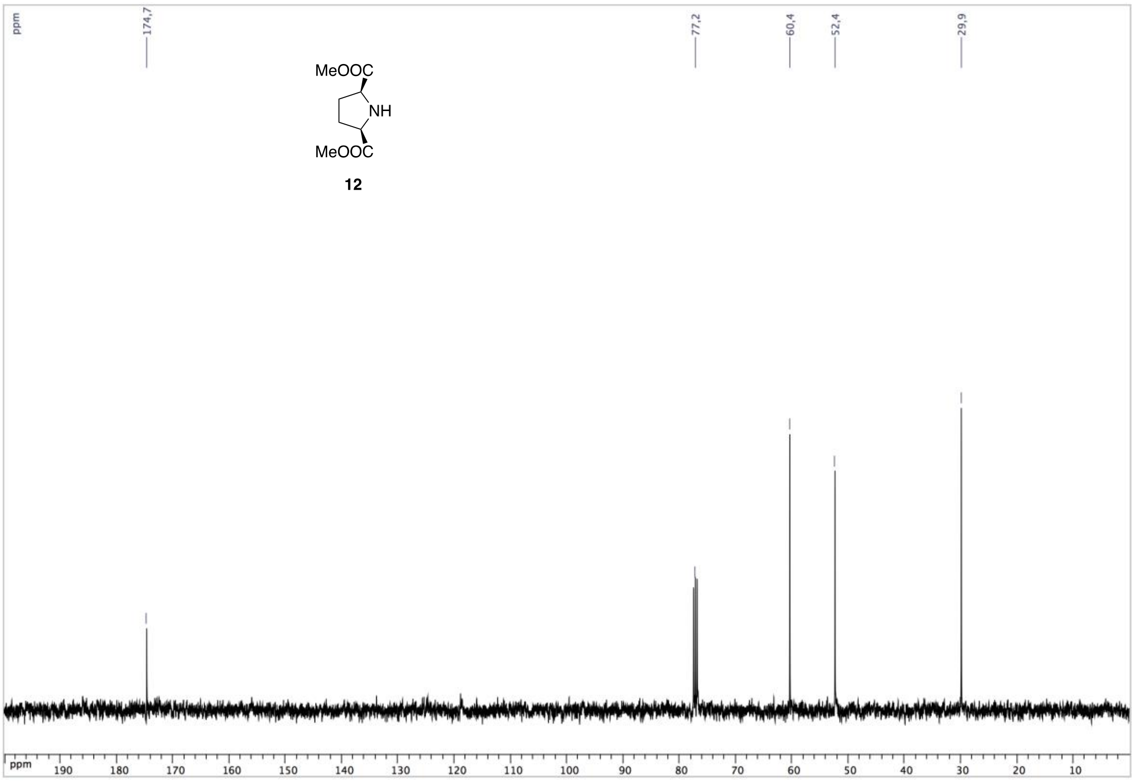
ppm
174,7
77,2
60,4
52,4
29,9
MeOOC
NH
MeOOC
12
ppm
190
180
170
160
150
140
130
120
110
100
90
80
70
60
50
40
30
20
10

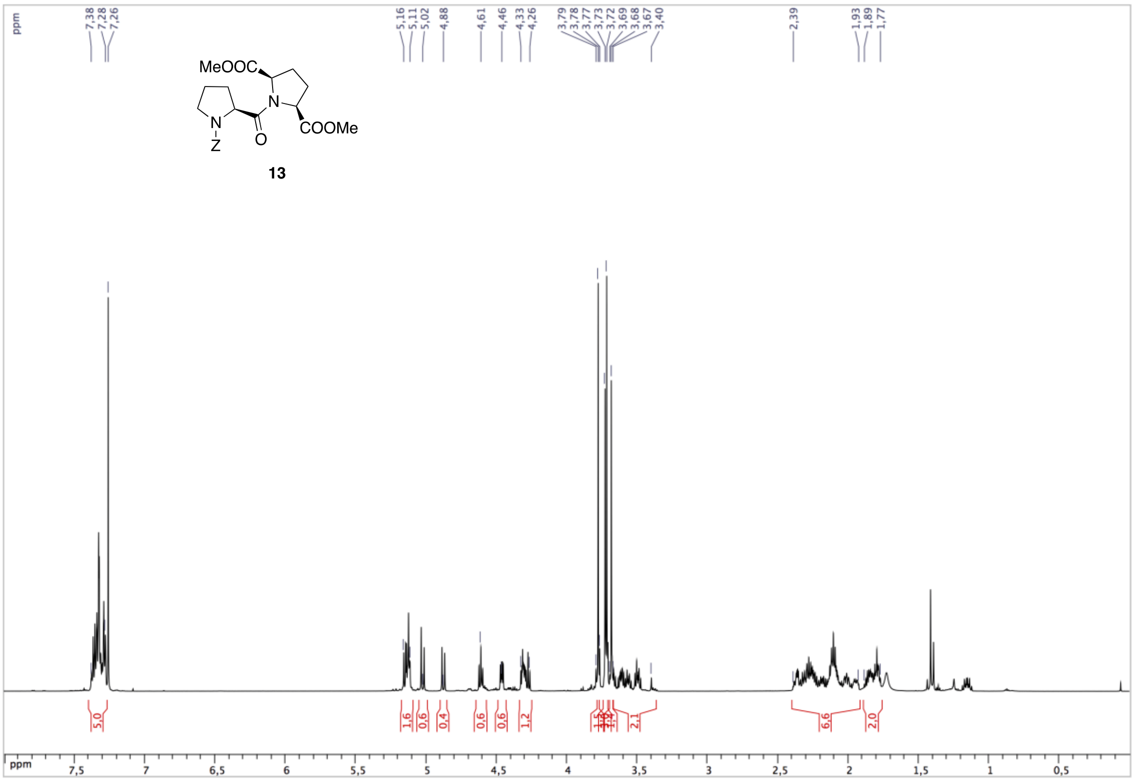
7,38
7,28
7,26
5,16
5,11
5,02
4,88
4,61
4,46
4,33
4,26
3,79
3,78
3,77
3,73
3,72
3,69
3,68
3,67
3,40
2,39
1,93
1,89
1,77
MeOOC
N
N
Z
O
COOMe
13
5,0
1,6
0,6
0,4
0,6
0,6
1,2
2,1
6,6
2,0
ppm
7,5
7
6,5
6
5,5
5
4,5
4
3,5
3
2,5
2
1,5
1
0,5

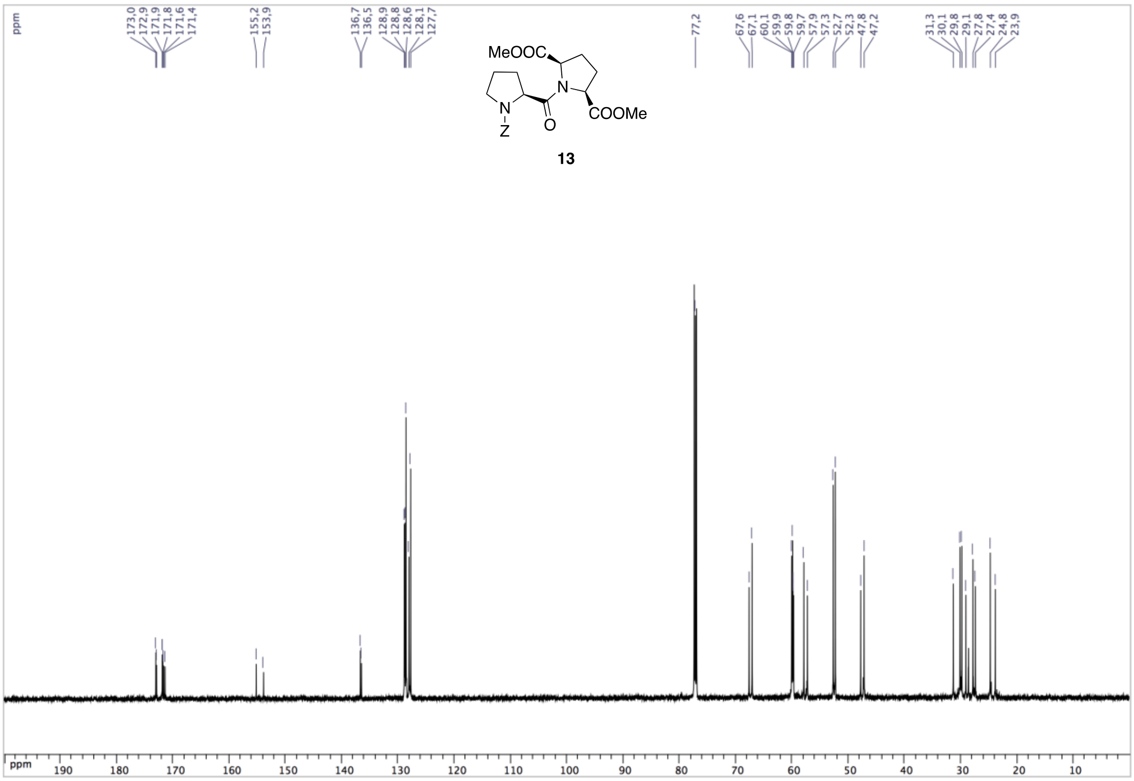
173,0
172,9
171,9
171,8
171,6
171,4
155,2
153,9
136,7
136,5
128,9
128,8
128,6
128,1
127,7
77,2
67,6
67,1
60,1
59,9
59,8
59,7
57,9
57,3
52,7
52,3
47,8
47,2
31,3
30,1
29,8
29,1
27,8
27,4
24,8
23,9
MeOOC
N
N
Z
O
COOMe
13
ppm
190
180
170
160
150
140
130
120
110
100
90
80
70
60
50
40
30
20
10

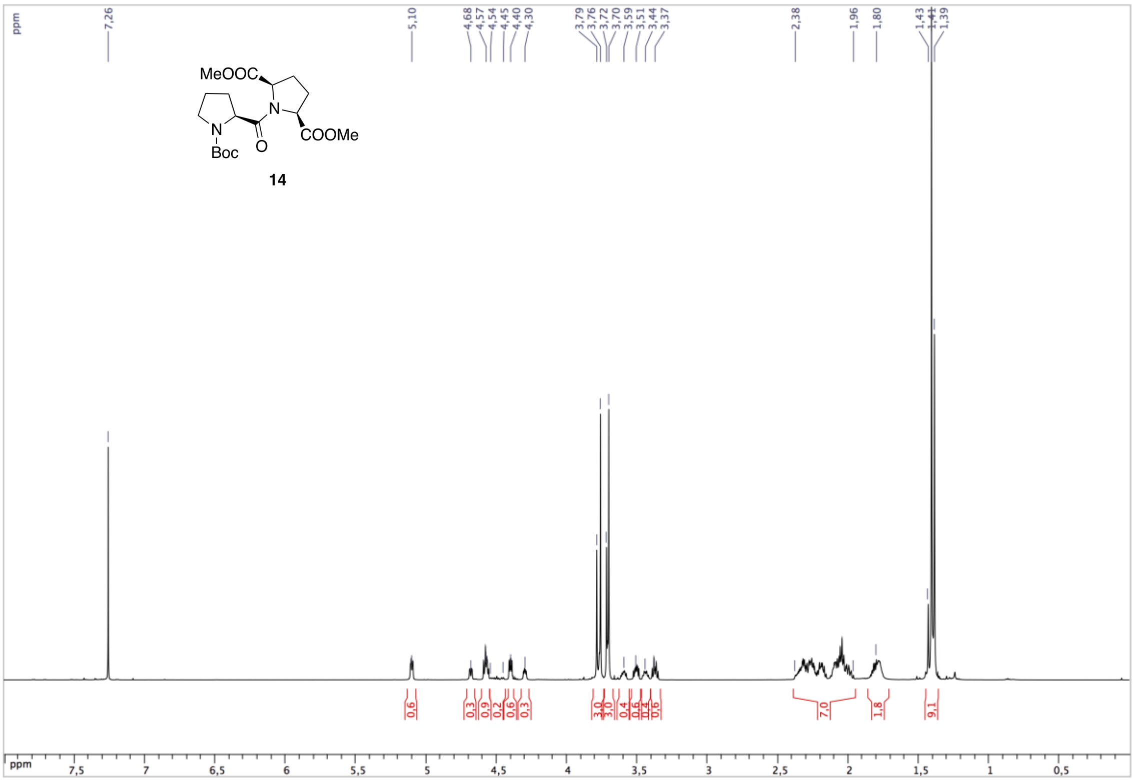
MeOOC
N
N
Boc
O
COOMe
14
ppm

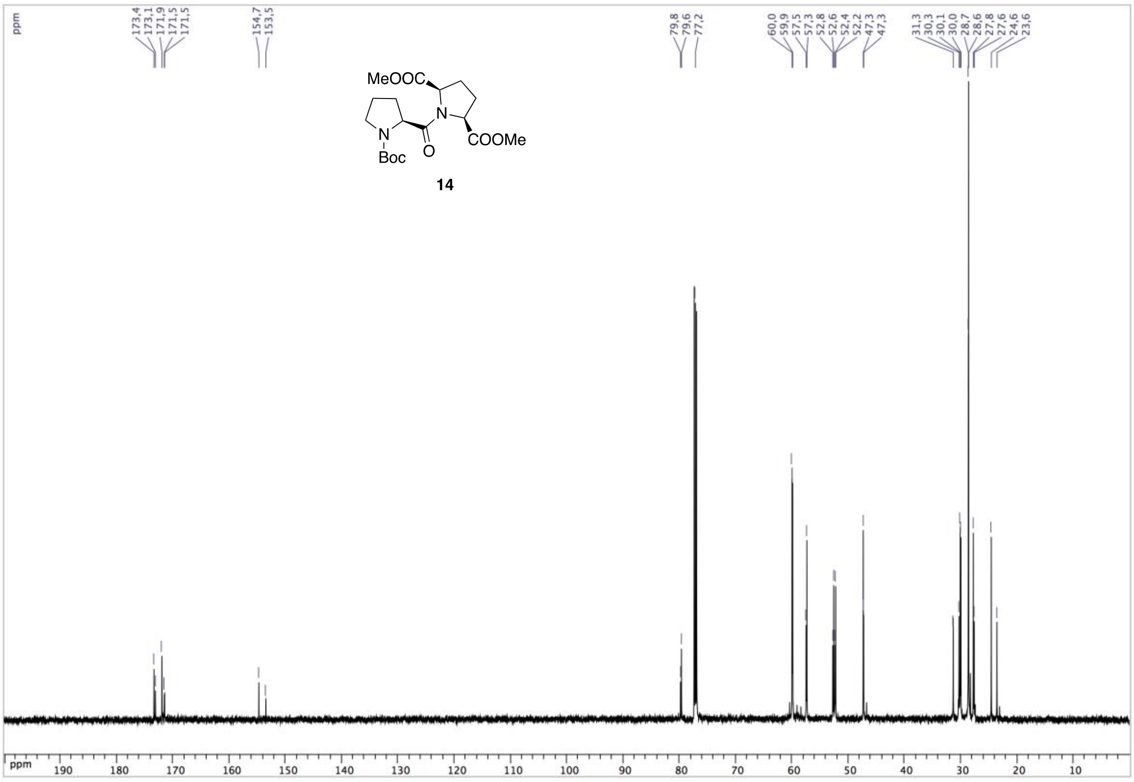
MeOOC
N
N
Boc
O
COOMe
14
ppm

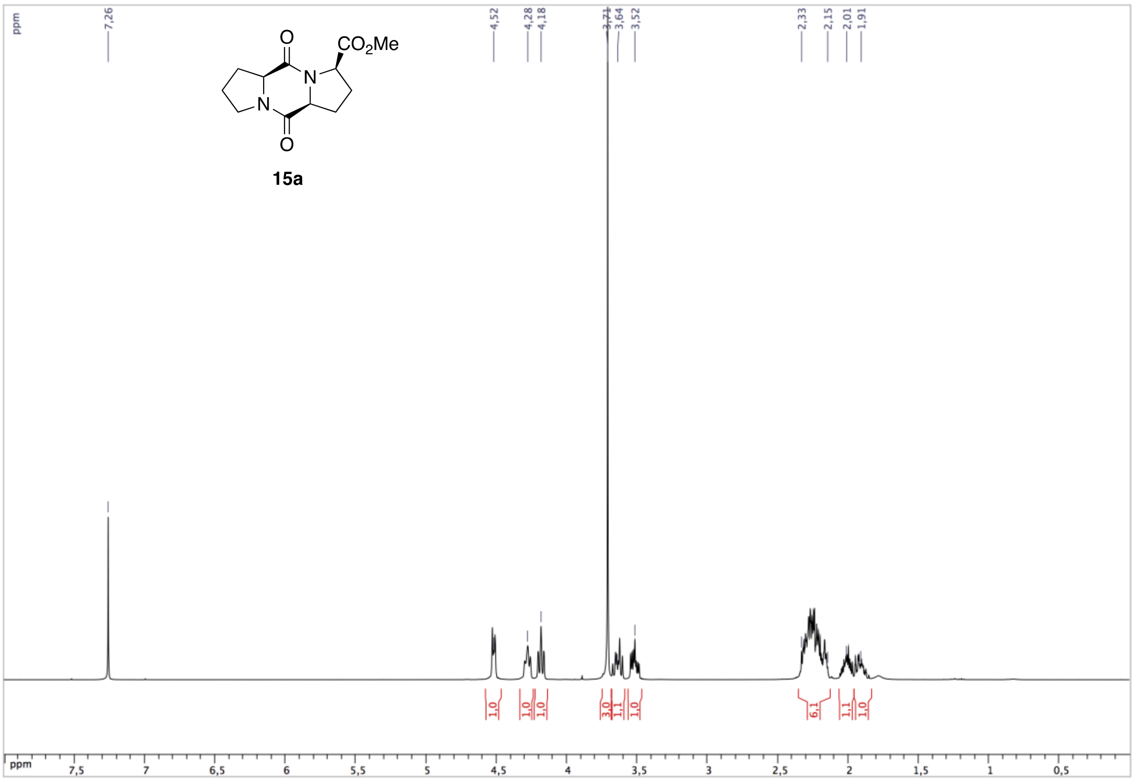
ppm
7,26
4,52
4,28
4,18
3,71
3,64
3,52
2,33
2,15
2,01
1,91
CO2Me
O
N
N
O
15a
1,0
1,0
1,0
3,0
1,1
1,0
6,1
1,1
1,0
ppm
7,5
7
6,5
6
5,5
5
4,5
4
3,5
3
2,5
2
1,5
1
0,5

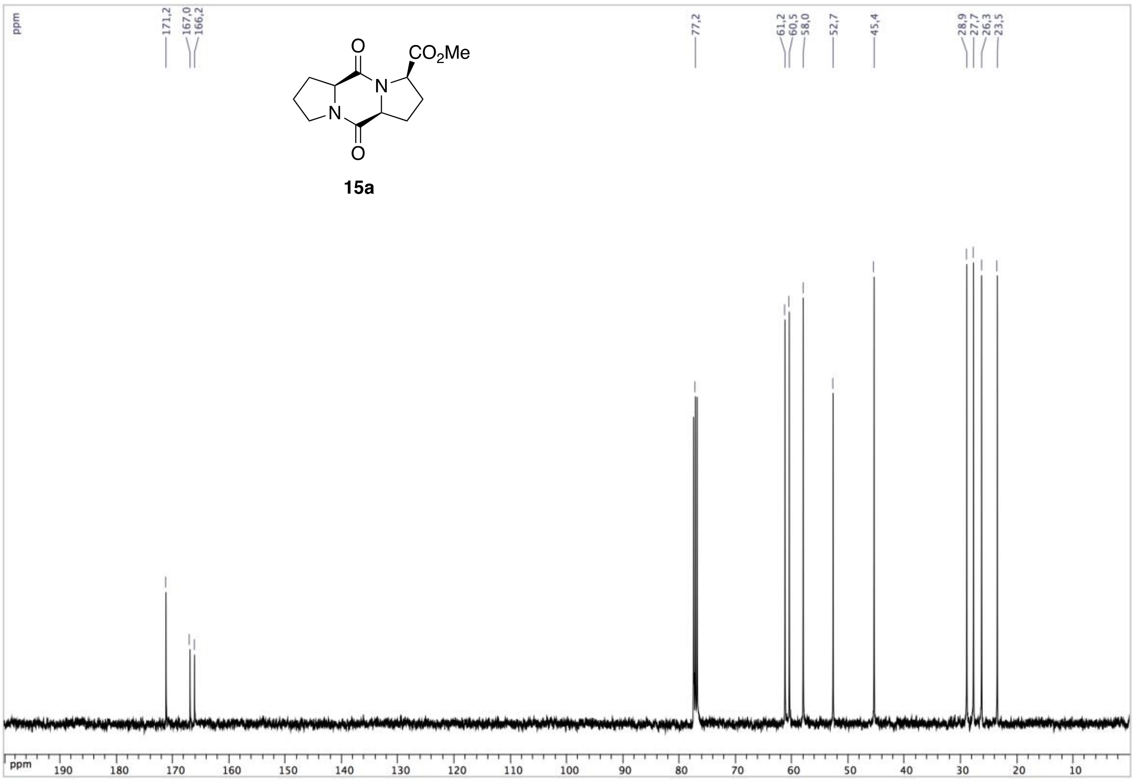
ppm
171,2
167,0
166,2
77,2
61,2
60,5
58,0
52,7
45,4
28,9
27,7
26,3
23,5
CO2Me
O
N
N
O
15a
ppm
190
180
170
160
150
140
130
120
110
100
90
80
70
60
50
40
30
20
10

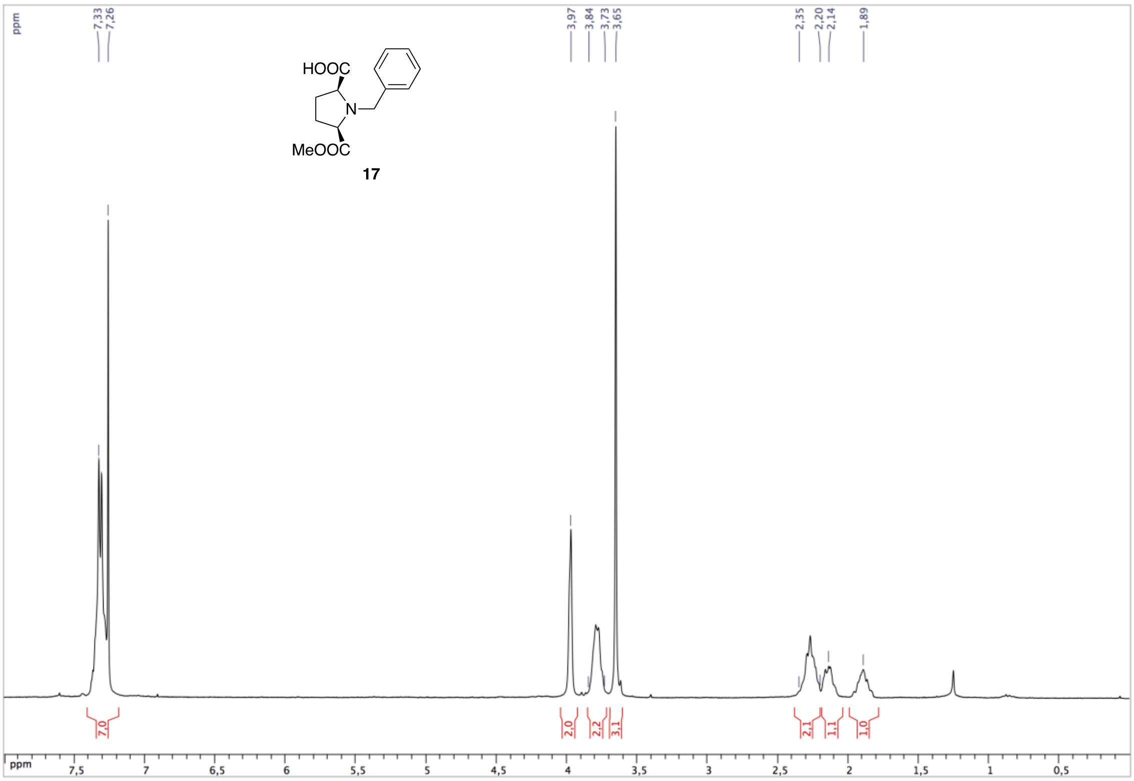
ppm
7,33
7,26
3,97
3,84
3,73
3,65
2,35
2,20
2,14
1,89
HOOC
N
MeOOC
17
7,0
2,0
2,2
3,1
2,1
1,1
1,0
ppm
7,5
7
6,5
6
5,5
5
4,5
4
3,5
3
2,5
2
1,5
1
0,5

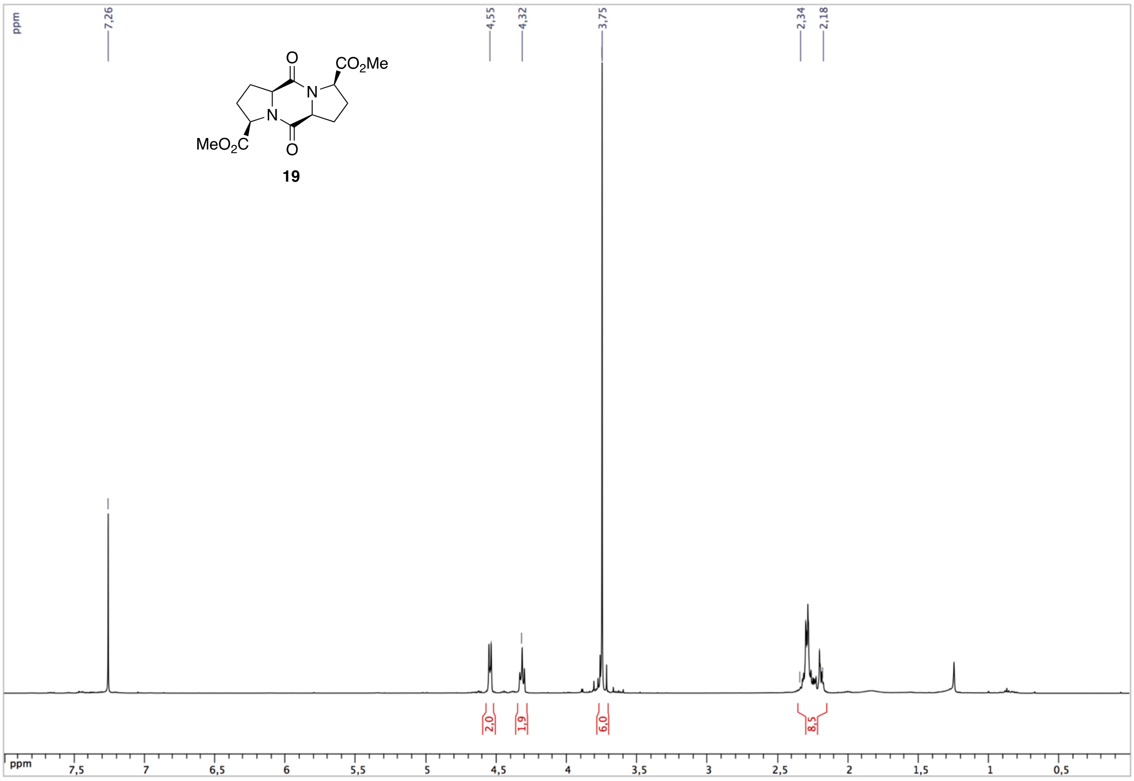
ppm
7,26
4,55
4,32
3,75
2,34
2,18
CO2Me
MeO2C
19
2,0
1,9
6,0
8,5
7,5 7 6,5 6 5,5 5 4,5 4 3,5 3 2,5 2 1,5 1 0,5

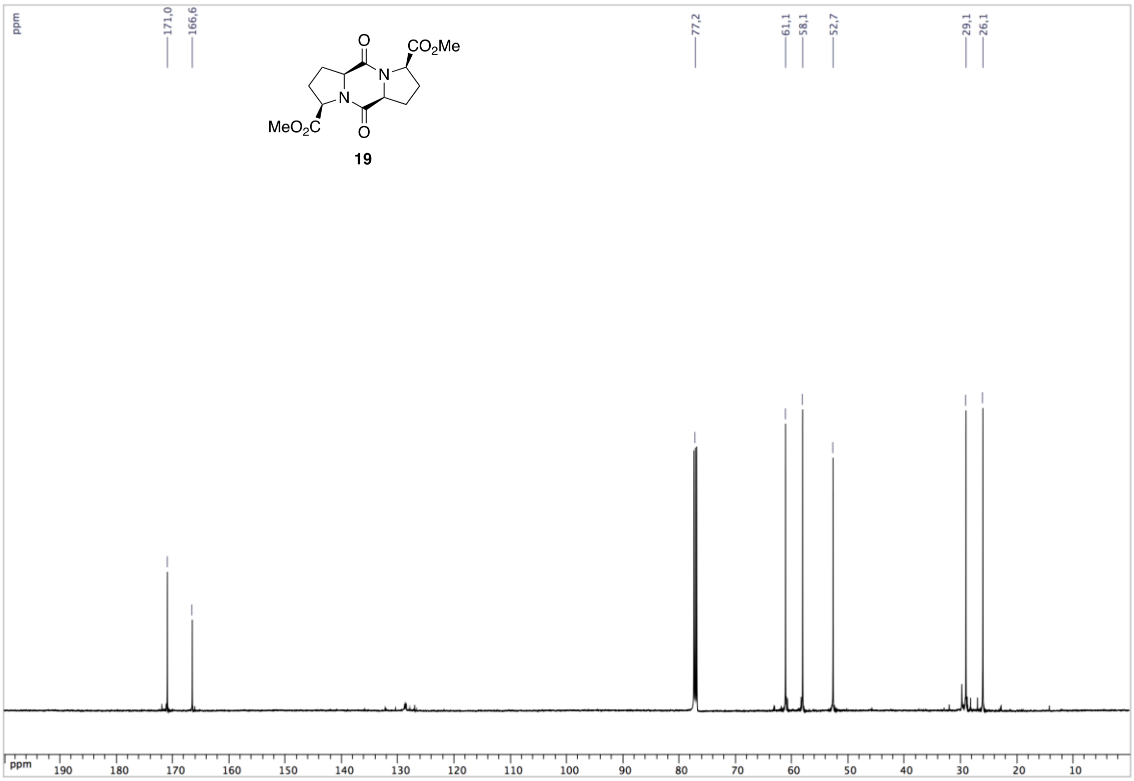
ppm
171,0
166,6
77,2
61,1
58,1
52,7
29,1
26,1
CO2Me
MeO2C
19
190 180 170 160 150 140 130 120 110 100 90 80 70 60 50 40 30 20 10

HPLC traces of compounds 3, 4, 7, 8 and 13

IBMM-UMR5247 - Laboratoire des acides aminés, peptides et protéines

Sample name: NIP190br
Data file: C:\Chem32\LC1220_000\Agilent1220_000\Data\NicolasPetry\NIP190br 2017-08-30 14-22-14.D
Description:
Injection volume: 5.000

Instrument: LC 1220
Injection date: 8/30/2017 2:23:48 PM
Acq. method: 0a100_3min_214nm.M

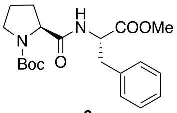

3

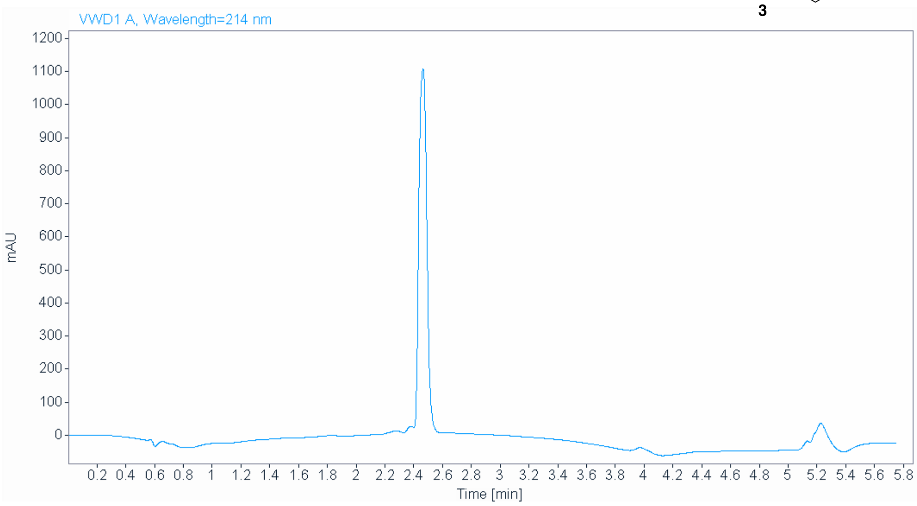

Signal: DAD1 A, Sig=254,4 Ref=off

RT [min]	Area	Height	Peak Area Percent

Signal: VWD1 A, Wavelength=214 nm

RT [min]	Area	Height	Peak Area Percent

**Sample name:**	NIP206br
**Data file:**	C:\Chem32\LC1220_000\Agilent1220_000\Data\NicolasPetry\NIP206br 2017-08-30 14-22-43.D
**Description:**	
**Injection volume:**	5.000
**Instrument:**	LC 1220
**Injection date:**	8/30/2017 2:31:05 PM
**Acq. method:**	0a100_3min_214nm.M

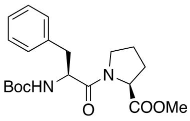

4

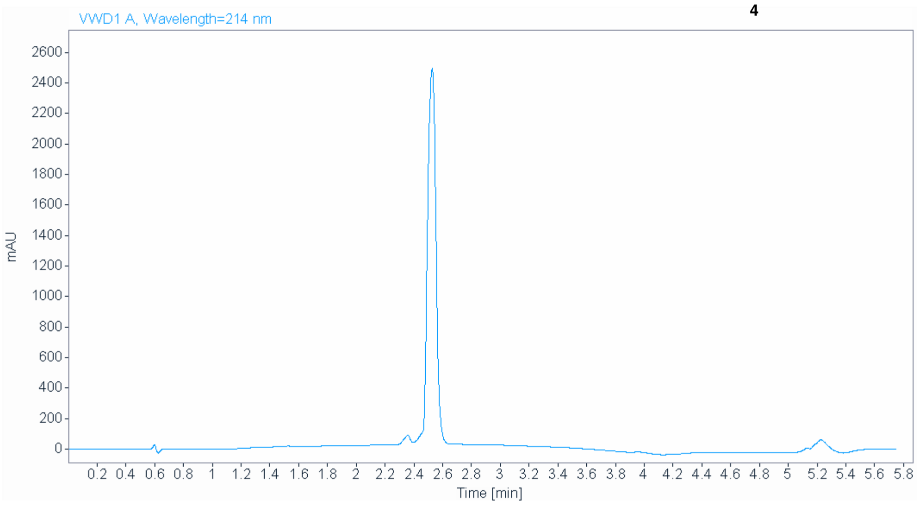

Signal: DAD1 A, Sig=254,4 Ref=off

RT [min]	Area	Height	Peak Area Percent

Signal: VWD1 A, Wavelength=214 nm

RT [min]	Area	Height	Peak Area Percent

IBMM-UMR5247 - Laboratoire des acides aminés, peptides et protéines

Sample name: NIP070br
Data file: C:\Chem32\LC1220_000\Agilent1220_000\Data\NicolasPetry\NIP070br 2017-08-30 13-03-26.D
Description:
Injection volume: 5.000

Instrument: LC 1220
Injection date: 8/30/2017 1:10:13 PM
Acq. method: 0a100_3min_214nm.M

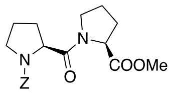

7

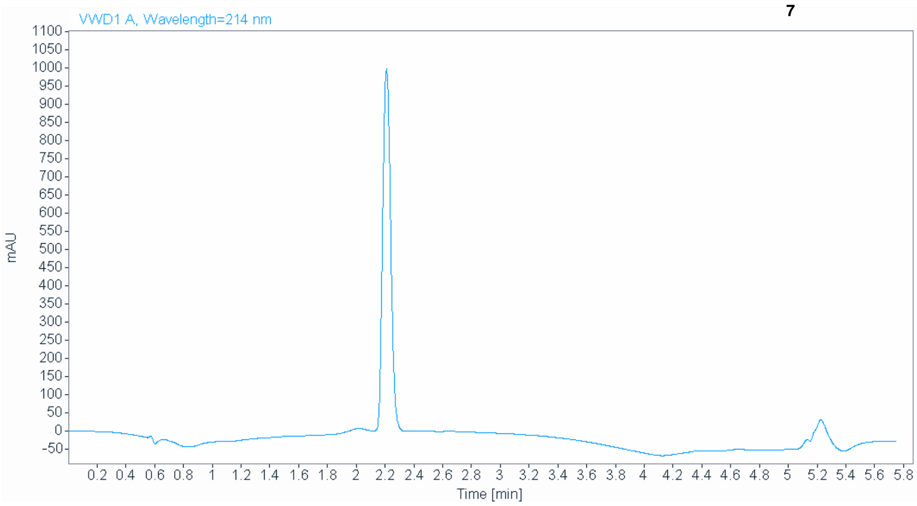

Signal: DAD1 A, Sig=254,4 Ref=off

RT [min]	Area	Height	Peak Area Percent

Signal: VWD1 A, Wavelength=214 nm

RT [min]	Area	Height	Peak Area Percent

IBMM-UMR5247 - Laboratoire des acides aminés, peptides et protéines

**Sample name:**	NIP094br
**Data file:**	C:\Chem32\LC1220_000\Agilent1220_000\Data\NicolasPetry\NIP094br 2017-08-30 13-03-42.D
**Description:**	
**Injection volume:**	5.000
**Instrument:**	LC 1220
**Injection date:**	8/30/2017 1:17:27 PM
**Acq. method:**	0a100_3min_214nm.M

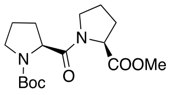

8

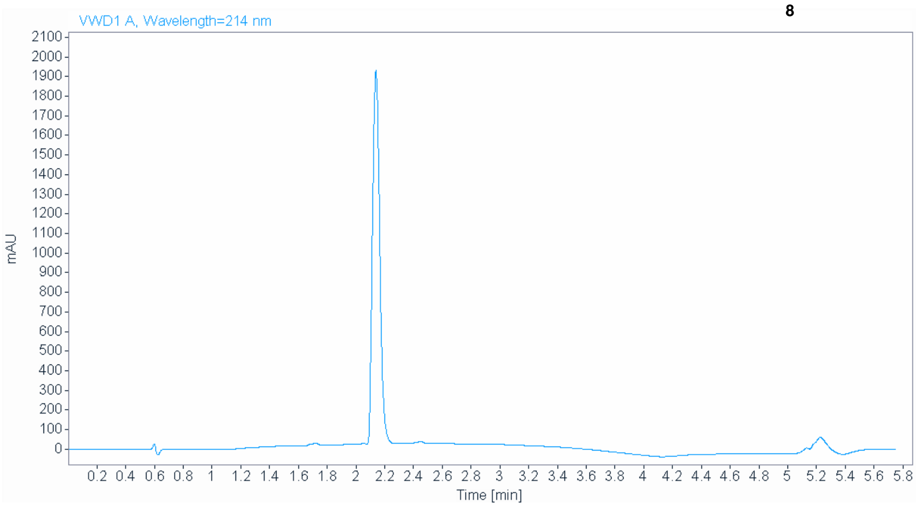

Signal: DAD1 A, Sig=254,4 Ref=off

RT [min]	Area	Height	Peak Area Percent

Signal: VWD1 A, Wavelength=214 nm

RT [min]	Area	Height	Peak Area Percent

IBMM-UMR5247 - Laboratoire des acides aminés, peptides et protéines

Sample name: NIP114br
Data file: C:\Chem32\LC1220_000\Agilent1220_000\Data\NicolasPetry\NIP114br 2016-07-08 09-21-25.D
Description:
Injection volume: 5.000

Instrument: Agilent1220
Injection date: 7/8/2016 9:24:17 AM
Acq. method: 0a100_3min_214nm.M

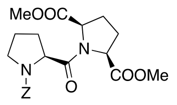

13

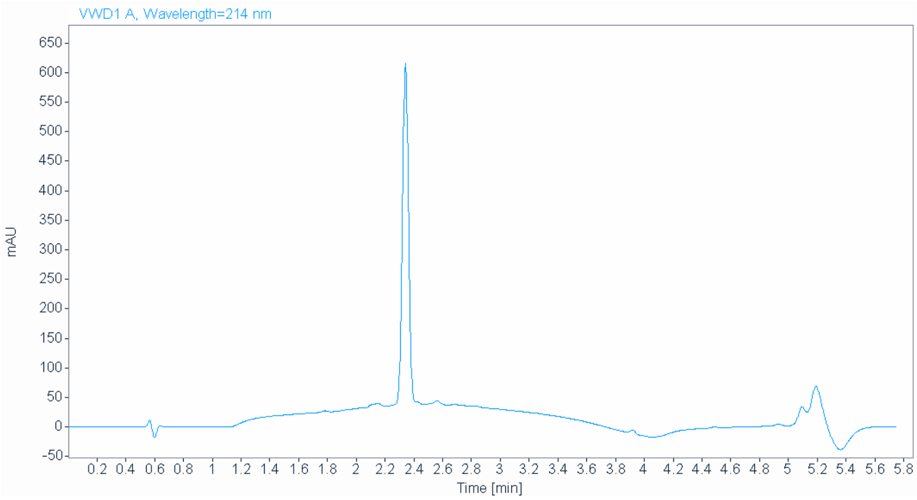

Signal: DAD1 A, Sig=254,4 Ref=off

RT [min]	Area	Height	Peak Area Percent

Signal: VWD1 A, Wavelength=214 nm

RT [min]	Area	Height	Peak Area Percent
