# Supplementary material for: A mechanochemical approach to access the proline–proline diketopiperazine framework
Source: Beilstein J Org Chem. 2017 Oct 19;13:2169–78. doi: 10.3762/bjoc.13.217 (PMC5669228; doi:10.3762/bjoc.13.217)
Supplement: File 2 — Crystallographic data. [file Beilstein_J_Org_Chem-13-2169-s002.pdf]

Supporting Information

for

A mechanochemical approach to access the proline–proline diketopiperazine framework

Nicolas Pétry[1], Hafid Benakki[1,2], Eric Clot[3], Pascal Retailleau[4], Farhate Guenoun[2], Fatima Asserar[2], Chakib Sekkat[2], Thomas-Xavier Métro*[1], Jean Martinez[1] and Frédéric Lamaty*[1]

Address: [1]Institut des Biomolécules Max Mousseron (IBMM), UMR 5247, CNRS, Université de Montpellier, ENSCM, Campus Triolet, Place Eugène Bataillon, 34095 Montpellier Cedex 5, France, [2]Laboratory of Chemistry Biology Applied to the Environment, Faculty of Sciences, Moulay Ismail University BP: 11201 Zitoune Meknès, Morocco, [3]Institut Charles Gerhardt, UMR 5253 CNRS-UM-ENSCM, Université de Montpellier, Place Eugène Bataillon, cc 1501, 34095 Montpellier Cedex 5, France and [4]Institut de Chimie des Substances Naturelles, CNRS UPR 2301, Université Paris-Saclay, 1 Avenue de la Terrasse, 91198 Gif-sur-Yvette, France

Email: Thomas-Xavier Métro - thomas-xavier.metro@umontpellier.fr; Frédéric Lamaty - frederic.lamaty@umontpellier.fr

*Corresponding author

Crystallographic data

Diffraction data were collected at room temperature on a RIGAKU XtaLabPro diffractometer equipped with a Mo microfocus sealed tube generator coupled to a double-bounce confocal Max-Flux® multilayer optic and a HPAD PILATUS3 R 200K detector for compound **10** and **15a**, or on a Rigaku MM007 HF copper rotating-anode generator equipped with Osmic confocal optics and a rapid II Curved Image Plate for compound **19**. Appropriate versions of the CrystalClear2 [1] were used according to the diffractometer to record and process the data. The structures were solved by new intrinsic phasing methods (SHELXLT)[2] and refined by full-matrix least-squares on F^2 values (SHELXL).[3] All heavy atoms were refined anisotropically. Hydrogen atoms were localized in difference electron density maps, but were refined isotropically with appropriate riding models. ORTEP-III [4] was used for structure presentation.
The *meso* form of the structure of compound **10** is confirmed around the crystallographic centre of inversion in the middle of the C1 - C1A bond, as previously shown by Feng and coll (2010)[5], CSD RefCode: WADCAH). Disorder is found in compound **15_a**: the methyl group displays two sets of methyl hydrogens that were refined with AFIX 123 and major part of occupancy refined to 0.58 (4) for C12, as well as the outer 5-membered ring that adopts in majority (occupancy factor of 0.65(2)) an envelope conformation on C2 whereas the minor conformation is similar to that of the opposite five-membered ring, whose closest pucker descriptor[6] is a twist on C3B–C4 (C7–C8, respectively). The absolute configuration (C1:S, C6:S and C9:R) is also determined based on significant Flack parameter[7] (–0.1(2)) and Bijvoet analysis using likelihood methods[8] (probability that the structure is inverted is only 0.2%) thanks to highly redundant good quality data albeit measured with a molly radiation. The same level of confidence can be applied to the compound **19,** which accommodates a two-fold rotation axis crossing the central diketopiperazine moiety orthogonally. The stereocentres C1 and C4 and those generated by the symmetry operation 1-x, y, 2-z are S and R respectively.

The crystallographic data for the structures reported in this paper have been deposited with the Cambridge Crystallographic Data Centre as supplementary publication. Copies of the data can be obtained free of charge on application to CCDC, e-mail: deposit@ccdc.cam.ac.uk.

X-ray data of compound **10**: $C_8H_{12}Br_2O_4$, M = 332.0 g/mol, monoclinic system, space group $P2_1/c$, a = 4.5421(4), b = 12.143(1), c = 10.5679(9) Å, β = 90.227(11) (9)°, Z = 2, V = 582.87(9) Å^3, Dc = 1.892 g·cm^{-3}, μ(Mo Kα) = 6.941 mm^{-1}, T = 293 K, crystal dimensions of 0.42 × 0.07 × 0.05 mm. The structure converged to the final R = 0.0431 and Rw = 0.0522 using 1331 independent reflections (θ_{max} = 27.56°). CCDC registration number 1552540.

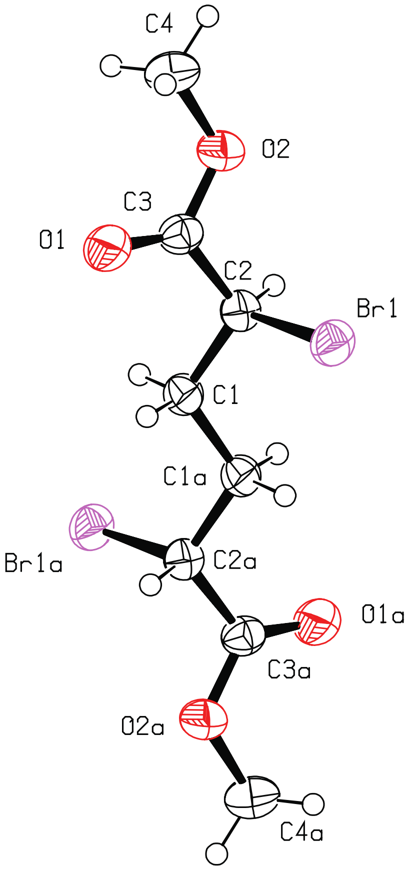

Figure S6. ORTEP projection of compound **10**, Displacement ellipsoids are drawn at the 50% probability level. Atoms labeled with the suffixes a are generated by the symmetry operation (1/2 - *x*, 3/2 - *y*, 1 - *z*).

X-ray data of compound **15_a**: $C_{12}H_{16}N_2O_4$, *M* = 252.27 g/mol, orthohombic system, space group $P2_12_12_1$, *a* = 5.9495(4), *b* = 9.7200(7), *c* = 21.3731(15) Å, Z = 4, V = 1215.21(15) Å^3, *Dc* = 1.379 g·cm^{-3}, μ(Mo Kα) = 0.104 mm^{-1}, *T* = 293 K, crystal dimensions of 0.31 × 0.08 × 0.06 mm. The structure converged to the final R = 0.0346 and Rw = 0.0925 using 2765 independent reflections (θ_{max} = 25.24°). CCDC registration number 1552541.

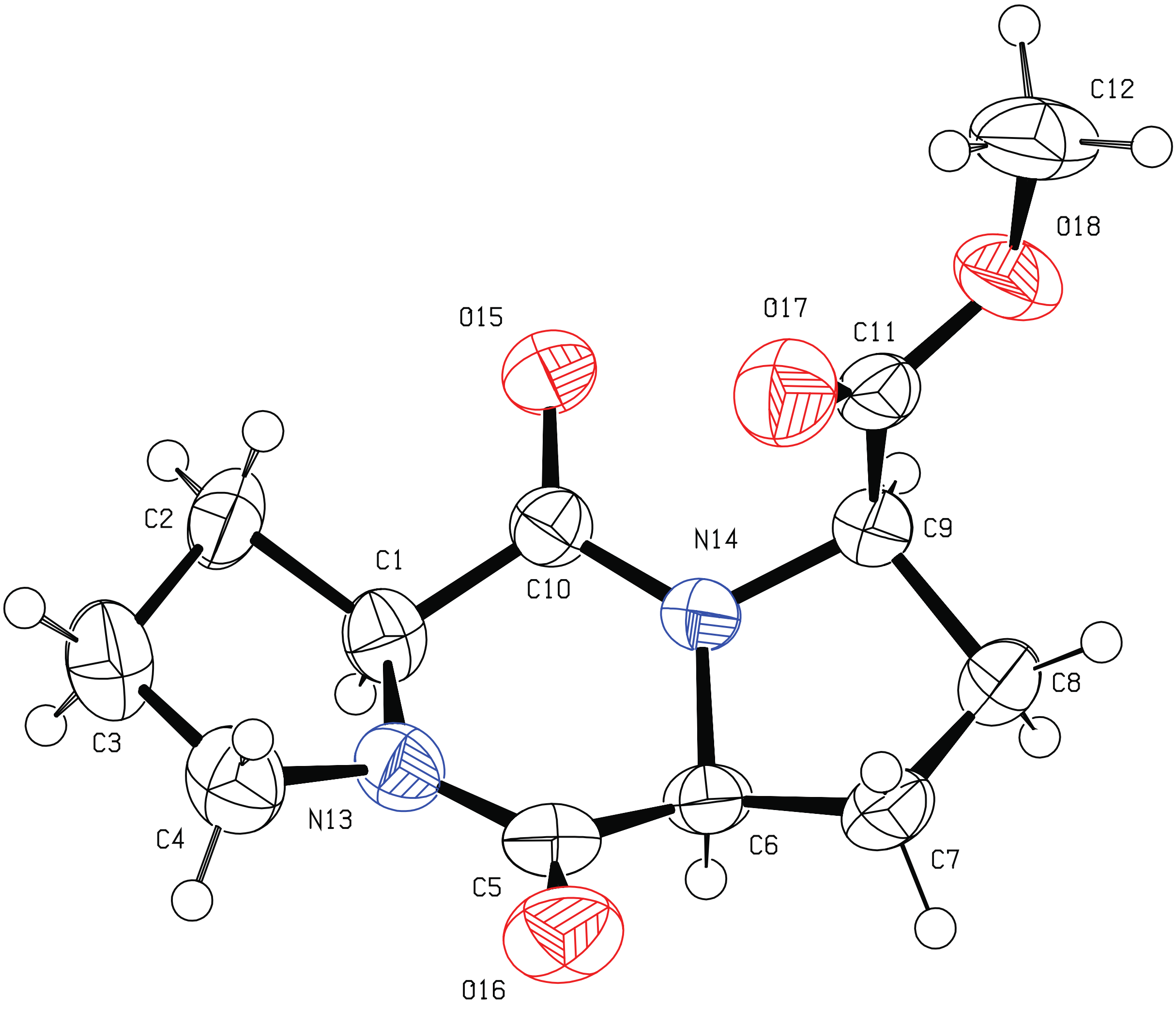

Figure S7. ORTEP projection of compound **15a**, ellipsoid probability 50%. No disorder is shown.

X-ray data of compound **19**: $C_{14}H_{18}N_2O_6$, *M* = 310.30 g/mol, monoclinic system, space group C2, a = 12.5371(10), b = 12.143(5), c = 10.5679(7) Å, β = 105.615(9)°, Z = 2, *V* = 737.75(10) Å^3, *D*c = 1.397 g·cm^{-3}, μ(Mo Kα) = 0.933 mm^{-1}, *T* = 293 K, crystal dimensions of 0.31 × 0.18 × 0.09 mm. The structure converged to the final R = 0.0462 and Rw = 0.0968 using 1339 independent reflections (θ_{max} = 67.68°). CCDC registration number 1552542.

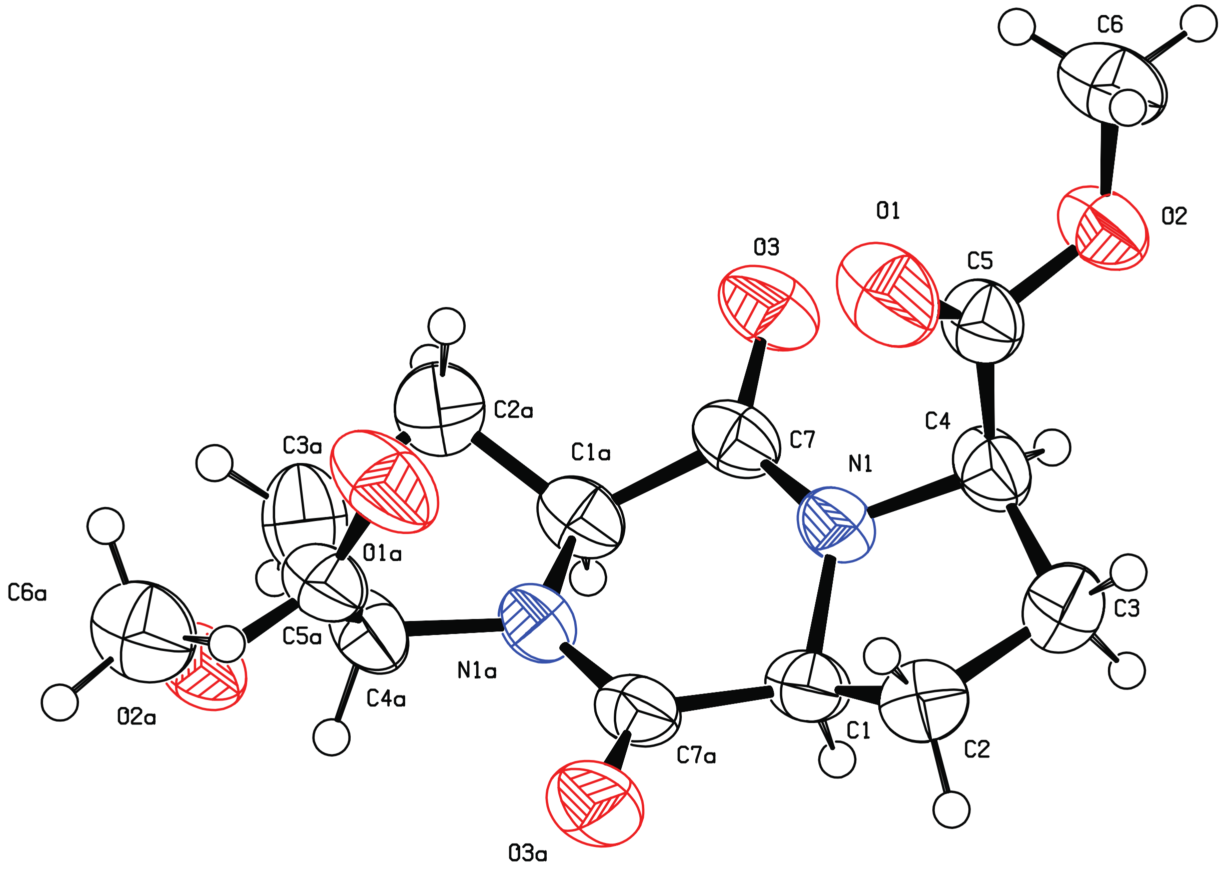

Figure S8. ORTEP projection of compound **19**, ellipsoid probability 50%

References for crystallographic data

(1) Rigaku (2012). *CrystalClear-SM Expert*. Rigaku Corporation, Tokyo, Japan.

(2) Sheldrick, G. M. (2015). *Acta Cryst.* A**71**, 3-8.

(3) Sheldrick, G. M. (2015). *Acta Cryst.* C**71**, 3-8.

(4) Burnett, M. N. & Johnson, C. K. (1996). *ORTEPIII*. Report ORNL-6895. Oak Ridge National Laboratory, Tennessee, USA.

(5) Feng, Z-Q., Ye, Y-F, Yang, X-L, Dong , Tand Huai-Qing Wang , H-Q. (2010) *Acta Cryst.*, E**66**, o3163.

(6) Cremer, D.& Pople, J.A. (1975), *J.Amer.Chem.Soc.*, 97, 1354-1358.

(7) Flack, H. D. (1983). *Acta Cryst.* A**39**, 876–881.

(8) Hooft, R. W. W., Straver, L. H. & Spek, A. L. (2008). *J. Appl. Cryst.* **41**, 96–103.
